# Host AMPK Is a Modulator of *Plasmodium* Liver Infection

**DOI:** 10.1016/j.celrep.2016.08.001

**Published:** 2016-08-25

**Authors:** Margarida T. Grilo Ruivo, Iset Medina Vera, Joana Sales-Dias, Patrícia Meireles, Nil Gural, Sangeeta N. Bhatia, Maria M. Mota, Liliana Mancio-Silva

**Affiliations:** 1 Instituto de Medicina Molecular, Faculdade de Medicina, Universidade de Lisboa, 1649-028 Lisboa, Portugal; 2 Department of Health Sciences and Technology, Massachusetts Institute of Technology, Cambridge, MA 02142, USA

## Abstract

Manipulation of the master regulator of energy homeostasis AMP-activated protein kinase (AMPK) activity is a strategy used by many intracellular pathogens for successful replication. Infection by most pathogens leads to an activation of host AMPK activity due to the energetic demands placed on the infected cell. Here, we demonstrate that the opposite is observed in cells infected with rodent malaria parasites. Indeed, AMPK activity upon the infection of hepatic cells is suppressed and dispensable for successful infection. By contrast, an overactive AMPK is deleterious to intracellular growth and replication of different *Plasmodium* spp., including the human malaria parasite, *P. falciparum*. The negative impact of host AMPK activity on infection was further confirmed in mice under conditions that activate its function. Overall, this work establishes the role of host AMPK signaling as a suppressive pathway of *Plasmodium* hepatic infection and as a potential target for host-based antimalarial interventions.

## Highlights

• *Plasmodium*-infected hepatic cells exhibit decreased AMPK activity
• AMPK suppression favors hepatic infection; its activation reduces parasite development
• AMPK activating compounds efficiently reduce liver infection in vitro and in vivo

## Graphical Abstract

**Figure:**
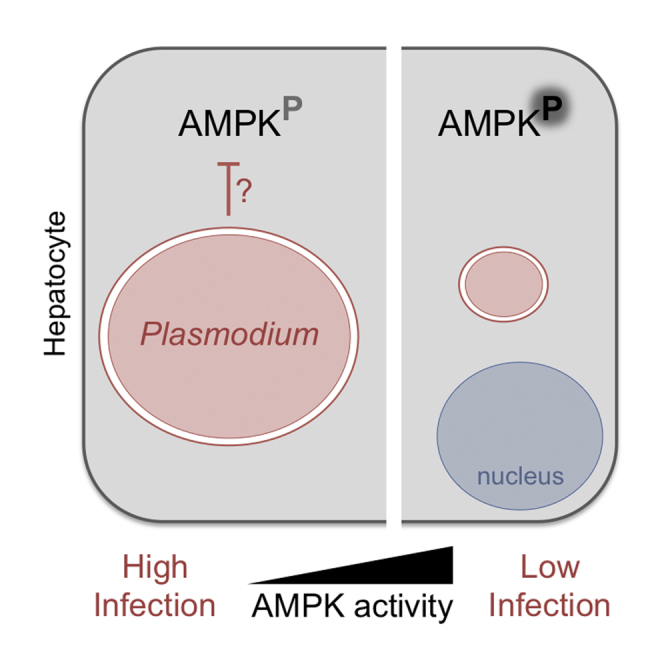

## Teaser

AMPK is a stress-activated kinase that regulates cellular energy homeostasis. Ruivo et al. show that AMPK signaling is relevant to hepatocyte infection by malaria parasites. Induction of host AMPK activity affects the ability of the host cell to support parasite growth in the liver, thus reducing the subsequent malaria burden.

## Introduction

*Plasmodium* spp. are obligate intracellular protozoan parasites and the etiological agents of malaria, an infectious disease that causes major morbidity and mortality and cripples socioeconomic growth. Lack of an effective vaccine and resistance to treatments are setbacks for controlling the disease (World Health Organization, 2015). Malaria infection begins in the liver, when the transmissive forms (sporozoites) invade and replicate by schizogony into thousands of new parasites (merozoites) inside hepatocytes. This high replicative capacity occurs within 48 hr in rodent parasites and up to 2 weeks in human parasites. Despite clear parasitism and subversion of host cell resources during hepatic infection, little is known about how *Plasmodium* infection modifies hepatocyte signaling. Previous transcriptional and post-transcriptional studies provide evidence of parasite-mediated alterations to host cell processes (Albuquerque et al., 2009, Kaushansky et al., 2013). Nonetheless, a comprehensive understanding of the hepatocyte response to this first stage of *Plasmodium* infection is needed to devise new antimalarial interventions.

Many intracellular pathogens actively alter host cellular metabolism as a strategy to produce optimal conditions for proliferation. An obvious metabolic target is AMPK (AMP-activated protein kinase), the master regulator of cellular energy homeostasis. AMPK is a conserved heterotrimeric (α catalytic, β and γ regulatory subunits) serine/threonine kinase that, as its name implies, responds to an increased AMP/ATP ratio. AMPK activation influences diverse pathways from glucose and lipid metabolism to cell-cycle regulation, promoting catabolism and inhibiting ATP consuming processes (reviewed in Hardie, 2014).

Manipulation of host AMPK activity is described for virus, bacteria, and parasite infections. For example, *Mycobacterium*, *Leishmania*, human cytomegalovirus, vaccinia, and simian vacuolating virus 40 induce AMPK activity, while hepatitis C virus (HCV) suppresses AMPK activity (Moreira et al., 2015, Singhal et al., 2014; reviewed in Brunton et al., 2013). Thus, AMPK modulation varies with the pathogen and the host cellular context and is dependent on the specific energetic requirements.

In this study, we investigated the role of host AMPK during the course of *Plasmodium* hepatic infection. We show that host AMPK function is suppressed during infection by these parasites. Using several in vitro and in vivo approaches, we demonstrate that activation of the AMPK signaling pathway impairs the intracellular replication of malaria liver-stage parasites.

## Results

### *Plasmodium* Hepatic Infection Leads to Decreased AMPK Function

**Figure 1:**
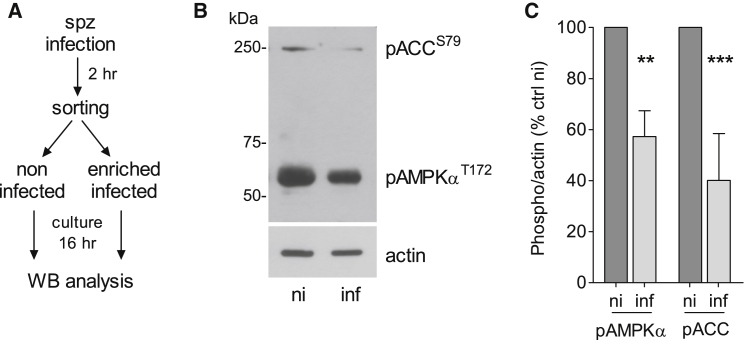
*P. berghei* Hepatic Infection Alters the AMPK Activation Status (A) Timeline of infection and sample collection. Huh7 cells were infected with GFP-expressing *P. berghei* sporozoites (spz) and subjected to fluorescence-activated cell sorting to separate infected from non-infected (ni) cells at 2 hr post-infection. Cells were re-plated 1:1 (infected:non-infected), cultured for 16 hr, and compared to non-infected by western blot (WB). (B and C) WB analysis of lysates from non-infected (ni) and enriched infected (inf) Huh7 cells collected at 18 hr post-infection, probing with anti-phospho-AMPKα (pAMPKα^T172^), -phospho-ACC (pACC^S79^), and -actin antibodies. (B) Representative blot and (C) quantitative analysis (mean ± SEM) of three independent experiments. Analysis of additional time points and control (ctrl) for total AMPKα abundance is shown in Figure S1. ^∗∗^p < 0.01; ^∗∗∗^p < 0.001.

AMPK activity can be determined by the phosphorylation of a threonine (T172) residue in the AMPKα catalytic subunit as well as the phosphorylation of the main downstream effector acetyl-coA (coenzyme A) carboxylase (ACC, S79), a rate-limiting enzyme in fatty acid synthesis (Hardie and Pan, 2002). To test whether AMPK activation is altered upon *Plasmodium* infection, we compared the phosphorylation status of AMPKα and ACC in non-infected Huh7 cells versus cells infected with the rodent parasite *P. berghei* (Figure 1A). Phosphorylation of AMPKα and ACC is lower in infected cells when compared to the non-infected cells at 18 hr post-infection (p < 0.01; Figures 1B and 1C). We confirmed a decrease in AMPKα phosphorylation over time (Figure S1A) and verified that total AMPKα abundance is not altered during infection (Figure S1B). A general reduction in phosphorylation was ruled out, since we observed a modest increase in phospho-Akt levels, as previously reported (data not shown; Kaushansky et al., 2013).

### Modulation of Host AMPK Affects *P. berghei* Hepatic Development In Vitro

**Figure 2:**
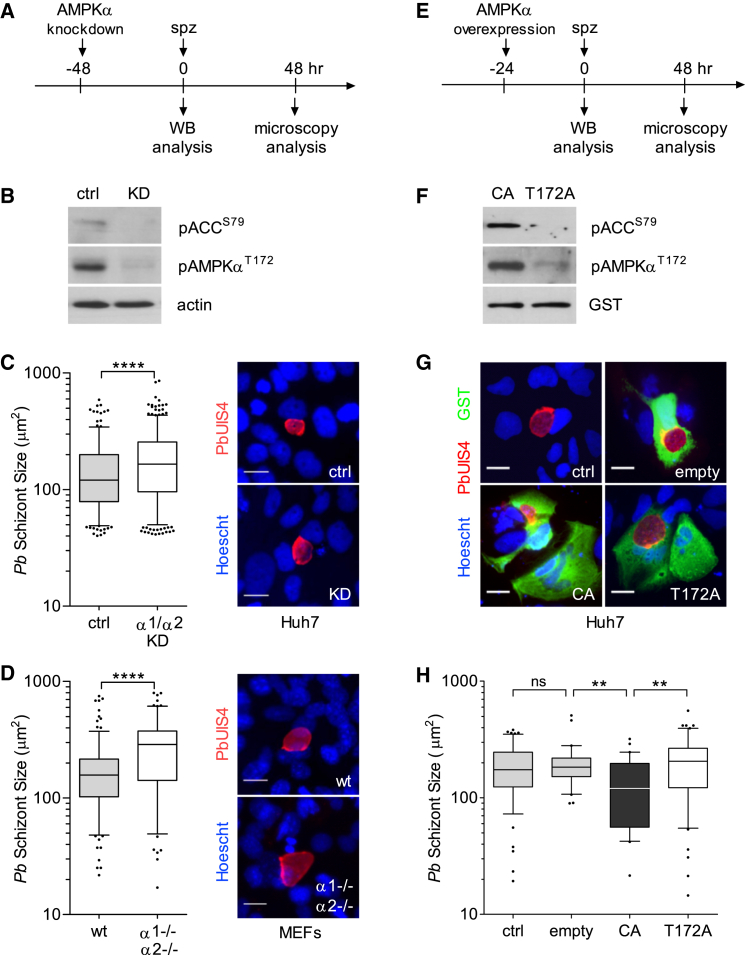
Modulation of Host AMPK Activity Alters *P. berghei* Development (A) Timeline of RNAi knockdown (KD) and infection. (B) pAMPKα^T172^ and pACC^S79^ status in lysates of Huh7 cells 48 hr after AMPK α1 and α2 KD. Representative blot of three independent experiments (KD efficiency, mean±SEM, 64.3% ± 10.3%). (C and D) Quantification of parasite size in AMPKα1/α2-depleted Huh7 cells (C) or AMPKα1^−/−^α2^−/−^ MEFs (D), assessed by microscopy at 48 hr post-infection. Parasite size is the area defined by staining with the parasite membrane marker PbUIS4, as shown in the representative images. Nuclei were stained with Hoechst. More than 100 parasites were imaged and analyzed for each of the three independent experiments. ctrl, control; wild type (WT). Scale bars, 20 μm. ^∗∗∗∗^p < 0.0001. The outliers in the boxplots represent 5% of data points. (E) Timeline of transfection with AMPKα1-carrying plasmids and infection. (F) Representative western blot of pAMPKα^T172^ and pACC^S79^ in lysates of Huh7 cells transfected with the truncated AMPKα1, constitutively active (CA), and mutated AMPKα1 (T172A) plasmids (see schematic of AMPKα1 domains and GST-tagged constructs in Figure S2A). GST was probed to detect transgenes. (G and H) Representative images (G) and quantification (H) of parasite size in cells expressing AMPKα1-CA, AMPKα1-T172A, or GST only (empty plasmid) in transfected or untransfected cells (ctrl). Transfected cells were identified with anti-GST antibodies, and parasites were detected with anti-PbUIS4. Nuclei were stained with Hoechst. Parasite size distribution in GST-negative cells is shown in Figure S2B. A representative of three independent experiments is shown (30–60 parasites examined per condition). The outliers in the boxplot represent 10% of data points. Scale bars, 20 μm. ^∗∗^p < 0.01; ns, non-significant.

Next, we investigated whether AMPK function could impact *P. berghei* infection. AMPKα catalytic subunit is encoded by two distinct genes, *prkaa1* (AMPKα1) and *prkaa2* (AMPKα2), which are expressed in hepatocytes. We knocked down both subunits by RNAi 48 hr prior to infection and confirmed a decrease in AMPKα and ACC phosphorylation at the time of infection (Figures 2A and 2B). Microscopic analysis of *P. berghei*-infected Huh7 cells at 48 hr post-infection revealed a small, but significant, increase in mean size distribution of schizont parasite forms (194 ± 127 μm^2^ versus 150.9 ± 99 μm^2^, p < 0.0001; Figure 2C). We confirmed this difference in parasite size by testing infection in mouse embryonic fibroblasts (MEFs) lacking both catalytic subunits (Laderoute et al., 2006) (291.2 ± 175 μm^2^ versus 176.8 ± 116 μm^2^, p < 0.0001; Figure 2D).

To test whether AMPK function might hinder infection, we overexpressed a constitutively active (CA) form of AMPKα1 subunit (Crute et al., 1998) in Huh7 cells. As controls, we expressed an inactive mutant AMPKα1 variant (T172A) and an empty plasmid (Figures 2E and S2A). AMPKα and ACC phosphorylation status was monitored by western blot analysis (Figure 2F). Microscopy examination at 48 hr post-infection revealed no significant difference in parasite size in cells not expressing the plasmids (Figure S2B). However, cells expressing the CA plasmid harbored significantly smaller hepatic schizonts, compared to controls (CA, 132.9 ± 83 μm^2^; T172A, 207.2 ± 119 μm^2^; and empty, 198.4 ± 92 μm^2^, p < 0.01; Figures 2G and 2H), implying that increased host AMPK activity decreases *P. berghei* hepatic growth.

### AMPK Agonists Restrict *Plasmodium* Hepatic Infection In Vitro

The impact of host AMPK activation during *P. berghei* infection was further characterized using a pharmacological approach. We exposed infected cells to known AMPK-activating compounds (salicylate, metformin, 2-deoxy-D-glucose, and A769662) (Hardie, 2014) (Table S1) and analyzed infection via luminescence and immunofluorescence assays in Huh7 cells (Figure S3). A dose-dependent reduction of total parasite load was observed for all tested compounds, with calculated half maximal effective concentration (EC_50_) values ranging from 200 μM to 1 mM (Figure S3A; Table S1), which are within or below the range described for other mammalian cell systems. Microscopy analysis revealed that AMPK-activating compounds led primarily to a significant decrease in schizont size, but not parasite numbers (Figures S3B and S3C).

**Figure 3:**
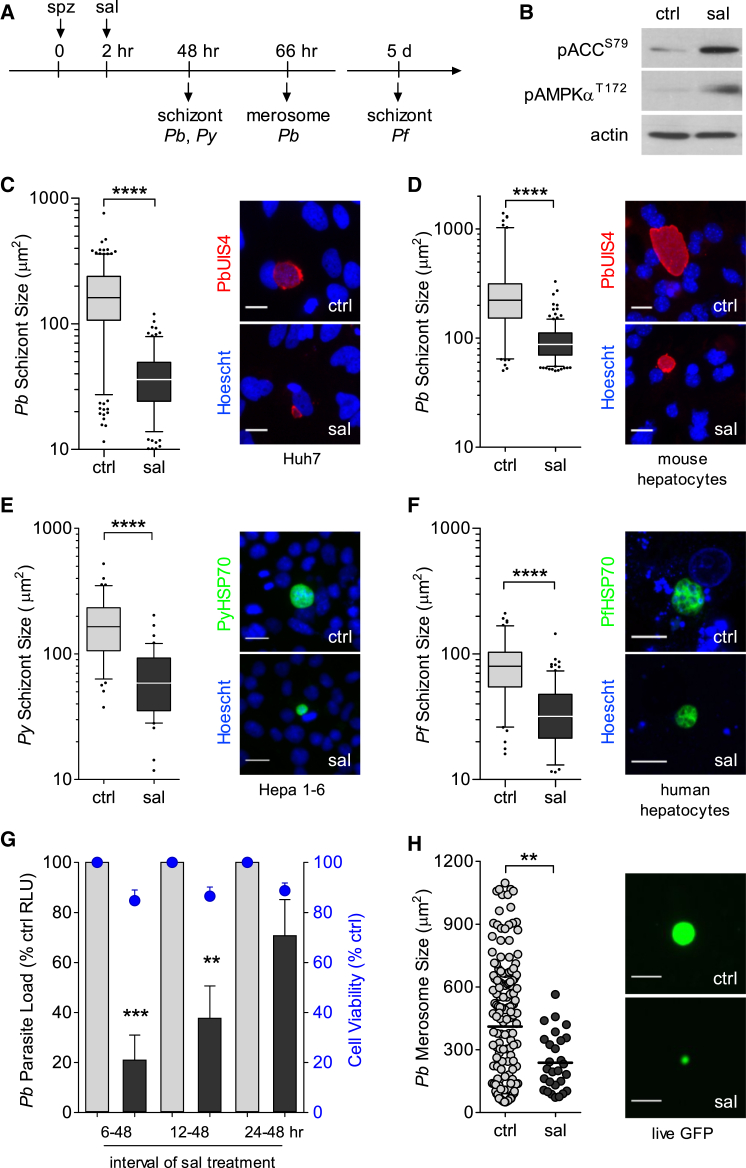
Pharmacological Activation of AMPK Reduces *Plasmodium* Infection (A) Timeline of infection and microscopy analysis upon treatment with salicylate (sal) or vehicle (ctrl, water) at 2 hr post-infection. Dose-dependent effects of salicylate and other AMPK agonists (metformin, 2-deoxy-D-glucose, and A769662) are shown in Figure S3. (B) Representative western blot of pAMPKα^T172^ and pACC^S79^ in lysates of non-infected Huh7 cells treated with salicylate (2.5 mM) for 24 hr. (C–F) Effect of salicylate treatment in Huh7 (C) or mouse primary hepatocytes (D) infected with *P. berghei* (*Pb*), Hepa1–6 cells infected with *P. yoelii* (*Py*) (E), and human primary hepatocytes infected with *P. falciparum* (*Pf*) (F). *Pb* and *Py*, 2.5 mM; *Pf,* 2 mM salicylate. Boxplots of parasite size distribution and illustrative images of three to four independent experiments are shown. Parasite size was determined based on the UIS4 or HSP70 signal after immunofluorescence assays. Nuclei were stained with Hoechst. *Pb* and *Py* scale bars, 20 μm; *Pf* scale bar, 10 μm. ^∗∗∗∗^p < 0.0001. (G) Time-course analysis of salicylate treatment (2.5 mM) starting at 6, 12, and 24 hr after infection of Huh7 cells with luciferase-expressing *P. berghei* parasites. Relative luminescence values (RLU) were measured at 48 hr. The bars are means ± SEM normalized to corresponding control, from three independent experiments. Cell viability (right y axis), measured by Alamar blue, is represented by the blue data points above each bar. ^∗∗^p < 0.01; ^∗∗∗^p < 0.001. (H) Size distribution scatterplot of detached merosomes from GFP-expressing *P. berghei*-infected HepG2 cells treated with salicylate (2.5 mM) from 2 to 66 hr. Data plotted is mean±SD, vehicle 411±311μm^2^, salicylate 239±135μm^2^. Data obtained from 3 independent experiments. Live GFP images of representative merosomes are shown. Bright-field images and quantification of detached merosome numbers are in Figures S4A and S4B. Data were obtained from three independent experiments. See Figure S4C for merosome analysis at later time points (66–74 hr) and Figure S4D for MSP1 staining at 66 hr. Scale bars, 50 μm. ^∗∗^p < 0.01.

To dissect the effect of host AMPK activation on parasite infection, we focused on salicylate, known to bind the AMPKβ1 subunit promoting AMPKα T172 phosphorylation (Hawley et al., 2012) (Figures 3A and 3B). The data show a similar negative effect on parasite development in Huh7 cells (40 ± 20.3 μm^2^ versus 177 ± 101.5 μm^2^, p < 0.0001; Figure 3C) and mouse primary hepatocytes infected with *P. berghei* (94.36 ± 36 μm^2^ versus 272.2 ± 209 μm^2^, p < 0.0001; Figure 3D), Hepa1-6 cells infected with *P. yoelii* (71 ± 42 μm^2^ versus 180.9 ± 106 μm^2^, p < 0.0001; Figure 3E), and human primary hepatocytes derived from different donors infected with *P. falciparum* (38 ± 23.9 μm^2^ versus 84 ± 40.9 μm^2^, p < 0.0001; Figure 3F). Thus, treatment with salicylate during hepatic infection leads to a reduction in parasite size, regardless of host cell or *Plasmodium* species.

To determine the time-course kinetics during which activated AMPK restricts parasite development, we exposed cells to salicylate at different time intervals post-infection. We observed that the parasite is most susceptible to salicylate treatment during the first 24 hr (Figure 3G). We then allowed *P. berghei* to fully mature in vitro under salicylate treatment into the final endstage of hepatic development, when merosomes containing fully mature merozoites are released from the substratum (66 hr; Sturm et al., 2006). First, we visualized the live GFP signal of detached merosomes from GFP-expressing *P. berghei*-infected cells at 66 hr and observed a reduction in merosome size (239 ± 135 μm^2^ versus 411 ± 311 μm^2^, p < 0.01; Figures 3H and S4A) and numbers (0.9 ± 0.9 per field versus 9 ± 4.5 per field, p < 0.0001; Figure S4B). Then, we examined luminescence levels from luciferase-expressing detached merosomes and observed an 80% reduction in total load up to 74 hr (p < 0.0001; Figure S4C), indicating that the decrease was not simply a delay in merosome release. Additionally, we performed immunofluorescence analysis with the merozoite surface marker (MSP1), essential for merozoite maturation, and observed that salicylate-treated cells contained smaller MSP1-positive schizonts (Figure S4D). The data demonstrate that AMPK agonists cause a reduction in parasite development during schizogony, with decreased release of merosomes, suggesting that the total number of merozoites reaching the blood to infect erythrocytes would be lower.

### AMPK Activation Reduces *P. berghei* Infection in Mice

**Figure 4:**
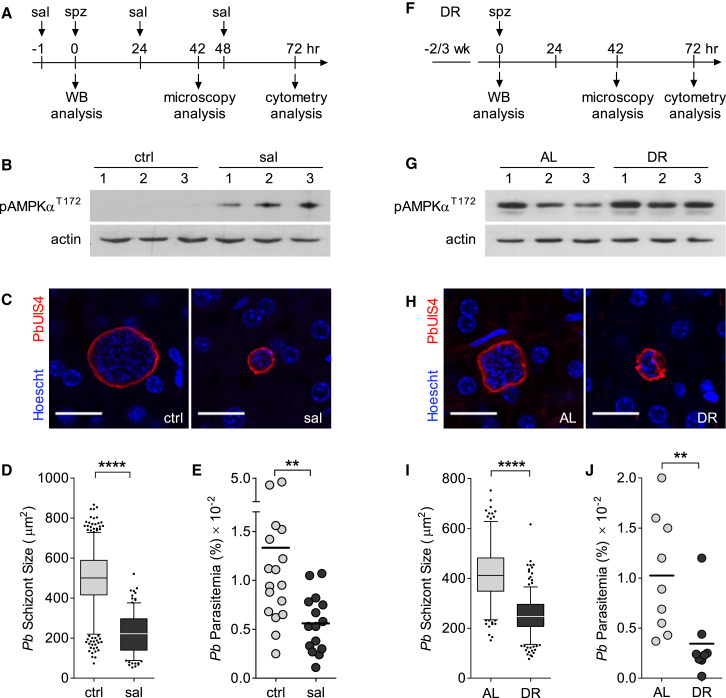
In Vivo Activation of AMPK Reduces Liver-Stage Infection (A–E) C57BL/6 mice treated with salicylate (sal, 300 mg/kg) or vehicle (ctrl, NaCl 0.9%). (F–J) C57BL/6 mice fed ad libitum (AL) or a dietary restriction (DR) regimen. Food intake and body weight changes are shown in Figures S5A and S5B. (A and F) Schedule of the treatments/diets, infections, and sample collection. (B and G) Western blot of pAMPKα^T172^ status in liver homogenates from non-infected mice 1 hr after injection of salicylate or vehicle (B) and non-infected mice on AL and DR diets (G). Quantification of pAMPKα^T172^ for AL and DR mice is given in Figure S5C. Numbers 1–3 represent individual mice. (C and H) Confocal representative images from infected livers. Scale bars, 20 μm. (D and I) Microscopy quantification of *P. berghei* size (area) in liver sections at 42 hr after infection. The parasite area was obtained after immunostaining with anti-PbUIS4 antibodies, as in Figures 2 and 3. Data were pooled from three mice per group (>100 parasites per mouse). The outliers in the boxplots represent 5% of data points.^∗∗∗∗^p < 0.0001. (E and J) Percentage of infected erythrocytes (parasitemia) measured by flow cytometry (≥8 mice per group) at 72 hr after infection. Data plotted is mean±SEM (×10^−2^), vehicle 1.33±0.28, salicylate 0.56±0.08, AL 1.03±0.19, DR 0.34±0.13. Data were pooled from two independent experiments. ^∗∗^p < 0.01.

Next, we asked whether our in vitro findings were relevant to an in vivo setting. First, we injected mice with salicylate to boost AMPK activity (Hawley et al., 2012) and confirmed increased AMPKα phosphorylation in mouse livers (Figures 4A and 4B). Then, mice were infected by intradermal injection of sporozoites, mimicking a natural mosquito bite. Parasite development under salicylate treatment mirrored the effects observed in vitro, with a significant reduction in size compared to control mice at 42 hr of infection (150.2 ± 110 μm^2^ versus 501.9 ± 35 μm^2^, p < 0.0001; Figures 4C and 4D). Next, we used flow cytometry to monitor the number of infected erythrocytes 72 hr after infection and observed a decrease in pre-patent parasitemia by 57% upon three doses of salicylate (p < 0.01; Figure 4E). A single dose was not sufficient to cause a significant reduction in parasitemia (data not shown).

As an alternative, we used a dietary restriction protocol, a method that activates AMPK via alterations in AMP/ATP ratios (Hardie, 2014). We restricted mice food intake by 30%–40% for 2–3 weeks prior and during liver-stage infection, leading to the expected body weight loss (Figures 4F, S5A, and S5B) and efficiently increased liver AMPK activation (Figures 4G and S5C). Physiological activation of AMPK resulted in a significant reduction of hepatic schizont size (252.8 ± 34 μm^2^ versus 399.6 ± 29 μm^2^, p < 0.0001; Figures 4H and 4I) and pre-patent blood stage infection (66% reduction, p < 0.01; Figure 4J), similar to salicylate treatment. Altogether, these results show that induction of host AMPK activity affects the ability of the host cell to support parasite growth in the liver, thus reducing the subsequent malaria burden.

## Discussion

The present study identifies host cell AMPK signaling as relevant to malaria liver-stage infection. We demonstrate that, while suppression of host AMPK favors *Plasmodium* hepatic infection, its activation has a negative impact on parasite growth. The results provide further insights into host hepatocyte signaling and reveal an emerging pattern where the host cell has increased Akt activity, decreased p53 (Kaushansky et al., 2013), and, as shown here, decreased AMPK activity. One advantage of such alterations in the infected cell is a metabolic state that supports rapid proliferation, known as the Warburg effect, a strategy that appears to be used by the parasite itself during schizogony, at least during erythrocytic stages (Salcedo-Sora et al., 2014).

Suppression of AMPK during hepatocyte infection may create a permissive environment serving multiple purposes, for example, through the inhibition of host autophagy (Kim et al., 2011), which may lead to parasite elimination. Alternatively, inhibition of AMPK and downstream targets (e.g., ACC) may help maintain the host cell biosynthetic capacity to sustain massive parasite replication. Indeed, *Plasmodium* is auxotrophic for certain metabolites, such as cholesterol (Labaied et al., 2011) and lipoic acid (Deschermeier et al., 2012), and scavenges host-derived phosphatidylcholine from hepatocytes (Itoe et al., 2014). A halt in cholesterol and fatty acid synthesis and breakdown, when AMPK is chronically activated, could have a negative impact on parasite growth. Such a mechanism has been described for HCV and Rift Valley Fever virus infections (Mankouri et al., 2010, Moser et al., 2012).

How are the levels of active AMPK lowered and maintained low during infection? This process can be a coping response from the host cell to the invading pathogen or a process prompted by the parasite. *Plasmodium* may actively promote inactivation of AMPK via its own effector molecules or indirectly through modulation of other host cell signaling pathways, leading to decreased AMPK function. As a member of the phylum Apicomplexa, *Plasmodium* sporozoites possess specialized organelles (micronemes and rhoptries) that secrete and inject molecules into host cells during invasion (Kemp et al., 2013). Furthermore, *Plasmodium* is also known to transport proteins beyond the parasite confines during intracellular hepatic growth (Kalanon et al., 2016, Singh et al., 2007). Alternatively, the sporozoite, known to traverse several hepatocytes before final invasion (Mota et al., 2001, Risco-Castillo et al., 2015), may establish infection in a cell with pre-existing low AMPK activity. Whether malaria sporozoites select to home in a cell with suppressed AMPK or modulate host AMPK activity via secretion/transportation of parasite-derived effector molecules remains to be determined.

AMPK activation via small-molecule treatment has been extensively studied, as clinically available drugs (salicylate and metformin) are widely used for treating conditions such as inflammation and diabetes, and are now being evaluated for their anti-tumorigenic properties (Hardie, 2014). Our results demonstrate that salicylate treatment of hepatocytes infected with rodent and human malaria parasites results in reduced parasite replication, which was also shown in vivo with *P. berghei* and is consistent with the effect of overexpressing a constitutively active AMPK in vitro. One caveat of using small molecules to induce AMPK activity is the possible lack of specificity. Salicylate, for example, at high doses has been described to uncouple mitochondria respiration and inhibit necrosis factor κB (NF-κB) signaling (Hawley et al., 2012, Steinberg et al., 2013). Thus, we cannot exclude that our observations with salicylate on parasite replication are fully AMPK dependent. Future experiments using liver-specific genetic mouse models of AMPK are necessary to assess the specificity of salicylate treatment or food restriction effect on liver infection. Furthermore, it would be worthwhile to investigate the impact of AMPK during *Plasmodium* infection of erythrocytes, where AMPK is important to regulate cell survival (Föller et al., 2009).

High energetic demands and auxotrophy by intracellular pathogens present a targetable approach to limit their growth. Drugs typically target pathogen-specific molecules, but due to the risk of selecting and spreading drug-resistant parasites, targeting of host molecules or pathways critical for successful pathogen development is an enticing strategy toward disease control. Host-based interventions have already been proposed against several pathogens, including hepatic and erythrocytic *Plasmodium* stages. For example, host p53 and Bcl-2 (Douglass et al., 2015), heme oxygenase 1 (Pena et al., 2012), erythrocyte G protein (Murphy et al., 2006), and MEK kinases (Sicard et al., 2011) have been suggested as potential targets. This concept is particularly valuable in the context of co-infections where multiple diseases could be tackled at once. The results presented here reveal the host AMPK as a druggable target with the potential to be further explored for antimalarial chemoprophylaxis and/or combination therapies.

## Experimental Procedures

### Cells, Transfections, and Infections

Cells were infected by adding freshly dissected *P. berghei*, *P. yoelii*, or *P. falciparum* sporozoites and analyzed by immunofluorescence assay or luminescence assay for luciferase-expressing parasites. For AMPKα knockdown, siPOOLs antisense oligonucleotides directed against *prkaa1* and *prkaa2* were used (siTOOLs Biotech). For AMPKα1 overexpression, cells were transiently transfected with pEBG-AMPKα1 plasmid (27632, Addgene) prior to infection.

### Mice, Diets, and Treatments

Male C57BL/6 mice were grouped based on body weight, housed four to five per cage, and allowed free access to water and food, except for mice on dietary restriction, which were given daily 60%–70% of the food consumed by the control group. Salicylate treatment was performed by intraperitoneal injection. Mice infections were performed by intravenous (5 × 10^4^ spz per mouse) or intradermal (5 × 10^3^ spz per mouse) injections and analyzed by microscopy on extracted livers or by flow cytometry, respectively. All experiments in animals were approved by the animal ethics committee at Instituto de Medicina Molecular, Lisboa (Portugal) and performed according to national and European regulations.

### Statistical Analysis

Statistics were determined with a Student’s t or Mann-Whitney U test for comparisons between two conditions and a one-way ANOVA for comparisons involving three or more conditions. Statistical significance was considered for p values below 0.05. The outliers in the boxplots represent 5%–10% of data points. Values in bar graphs are means ± SEM, and data mentioned in the text are means ± SD.

## Author Contributions

Conceptualization, M.M.M. and L.M.-S.; Investigation, M.T.G.R., I.M.V., J.S.-D., P.M., N.G., and L.M.-S.; Writing – Original Draft, M.T.G.R., I.M.V., and L.M.-S.; Writing – Review & Editing, M.T.G.R., I.M.V., M.M.M., and L.M.-S.; Funding Acquisition, M.M.M. and L.M.-S.; Supervision, S.N.B., M.M.M., and L.M.-S.

## Supplemental Information

Document S1. Supplemental Experimental Procedures, Figures S1–S5, and Table S1

Document S2. Article plus Supplemental Information
