# Supplementary material for: Host AMPK Is a Modulator of Plasmodium Liver Infection
Source: Cell Rep. 2016 Aug 25;16(10):2539–45. doi: 10.1016/j.celrep.2016.08.001 (PMC5014760; doi:10.1016/j.celrep.2016.08.001)
Supplement: Document S1. Supplemental Experimental Procedures, Figures S1–S5, and Table S1 [file mmc1.pdf]

Cell Reports, Volume *16*

Supplemental Information

Host AMPK Is a Modulator of *Plasmodium* Liver Infection

Margarida T. Grilo Ruivo, Iset Medina Vera, Joana Sales-Dias, Patrícia Meireles, Nil Gural, Sangeeta N. Bhatia, Maria M. Mota, and Liliana Mancio-Silva

SUPPLEMENTAL FIGURES

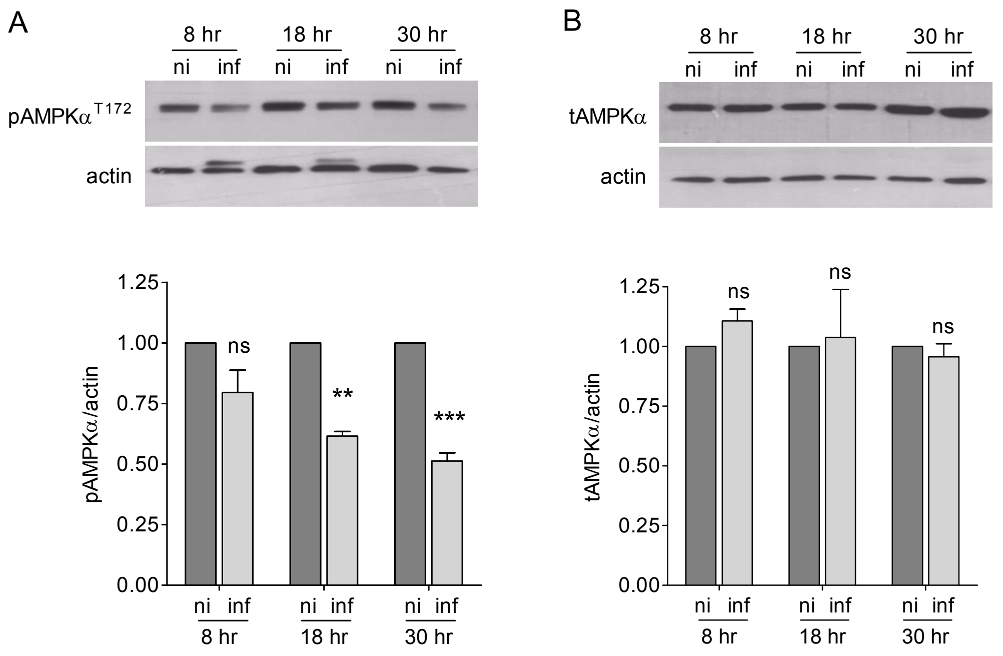

Figure S1. Related to Figure 1. Western blot analysis of lysates from *P. berghei* FACS-enriched infected (inf) and non-infected (ni) Huh7 cells harvested at 8, 18 and 30 hr post infection. Representative blots probing pAMPKα^{T172} (A) and total AMPKα (B). Stripping of the phospho antibody from the membranes was not efficient, therefore the same samples were loaded and analyzed in a separate membrane. Quantitative analysis of pAMPKα^{T172} and total AMPKα from 3 independent experiments (mean±SEM) is shown in the bottom panels. ns, non-significant; **$p<0.01$; ***$p<0.001$.

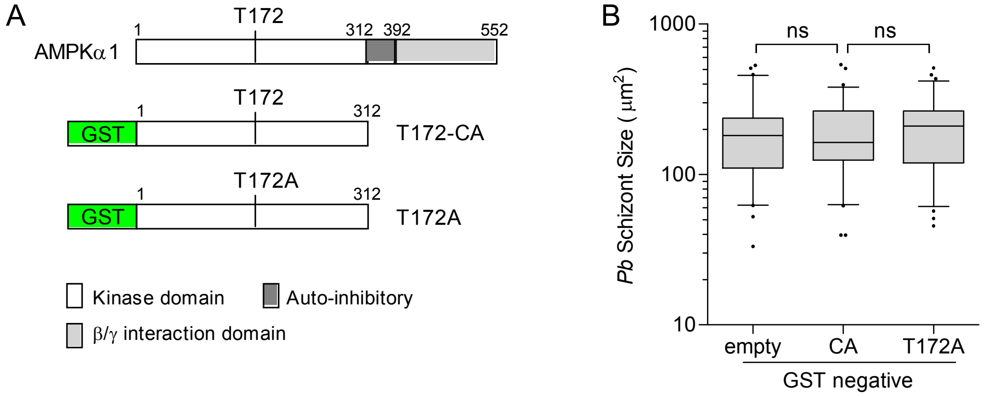

Figure S2. Related to Figure 2. (A) Schematic of the full-length AMPKα1 and its three domains: the catalytic domain (residues 1-312) containing the T172 phosphorylation site, the auto-inhibitory domain (312-392), and the C-terminal βγ interaction domain (392-552). Representation of the truncated constitutively active (CA) is shown below. The mutant construct (T172A) cannot be phosphorylated due to the replacement of the threonine to an alanine, and is used as negative control. The AMPKα1 CA and T172A proteins contain a N-terminal GST tag. (B) Quantification of parasite size (area) in Huh7 cells expressing the CA AMPKα1, T172A AMPKα1, or GST only (empty plasmid) in GST negative cells (50-70 parasites analyzed per condition). The mean±SD are as follows: 195.7±108.7μm^2, empty; 213.2±106.4μm^2, CA; 196.7±104.6μm^2, T172A. Graph shown is representative of 3 independent experiments. ns, non-significant.

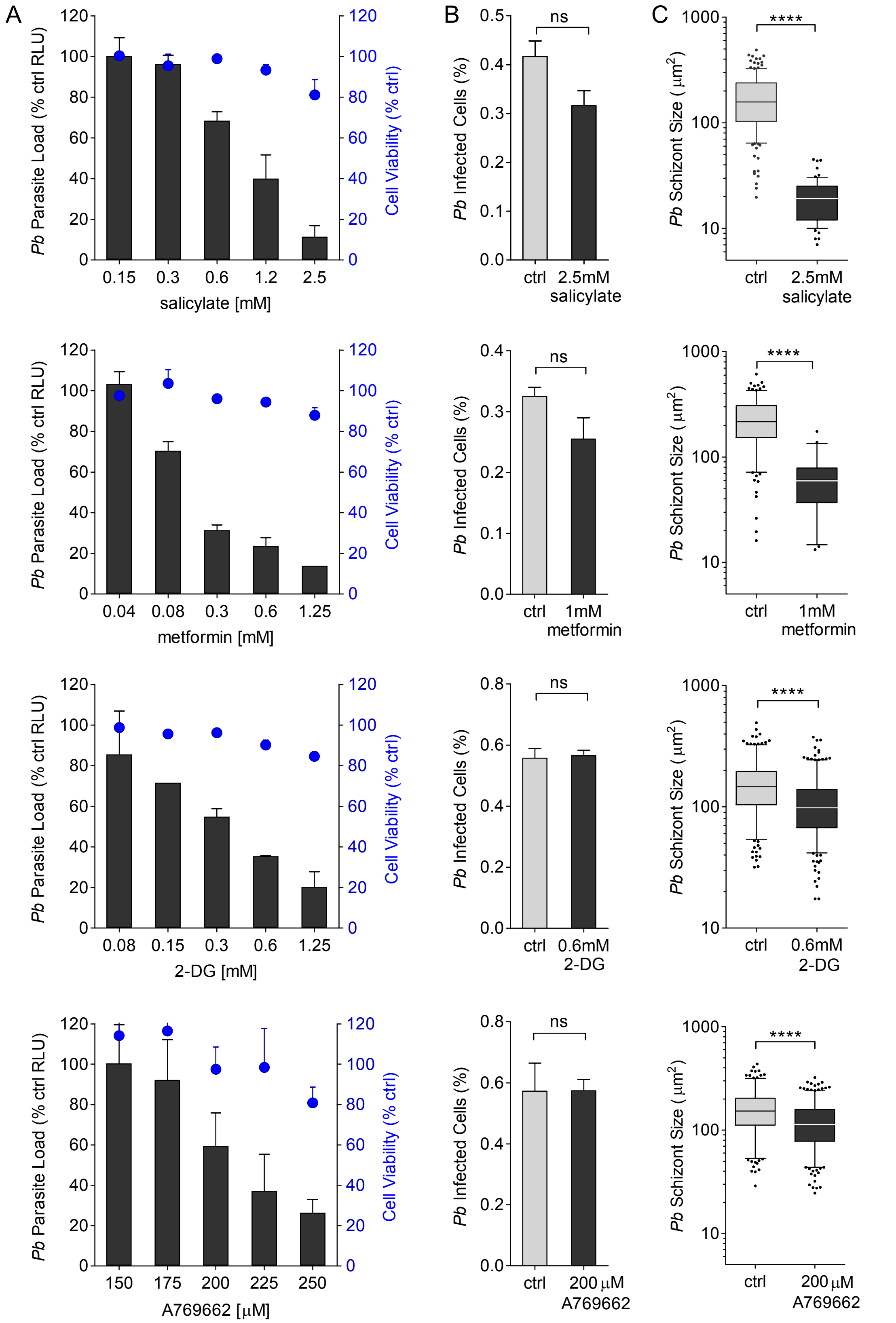

Figure S3. Related to Figure 3. Dose-dependent effect of various AMPK agonists: salicylate, metformin, 2-deoxy-D-Glucose (2-DG), and A769662 on *P. berghei* infection of Huh7 cells. (A) Parasite load at 48 hr post infection was measured via luminescence and is plotted as bar graphs (left y-axis) and host cell viability (right y-axis, blue) is plotted as blue data points above each bar. Values are mean±SEM from 2-3 independent experiments. EC_{50} values are in Table S1 and were determined by GraphPad Prism using non-linear regression variable slope (normalized) analysis. (B-C) Quantification of luciferase-expressing *P. berghei* schizont numbers (B) and size (C) by microscopic analysis. Data are representative of 2-3 independent experiments.

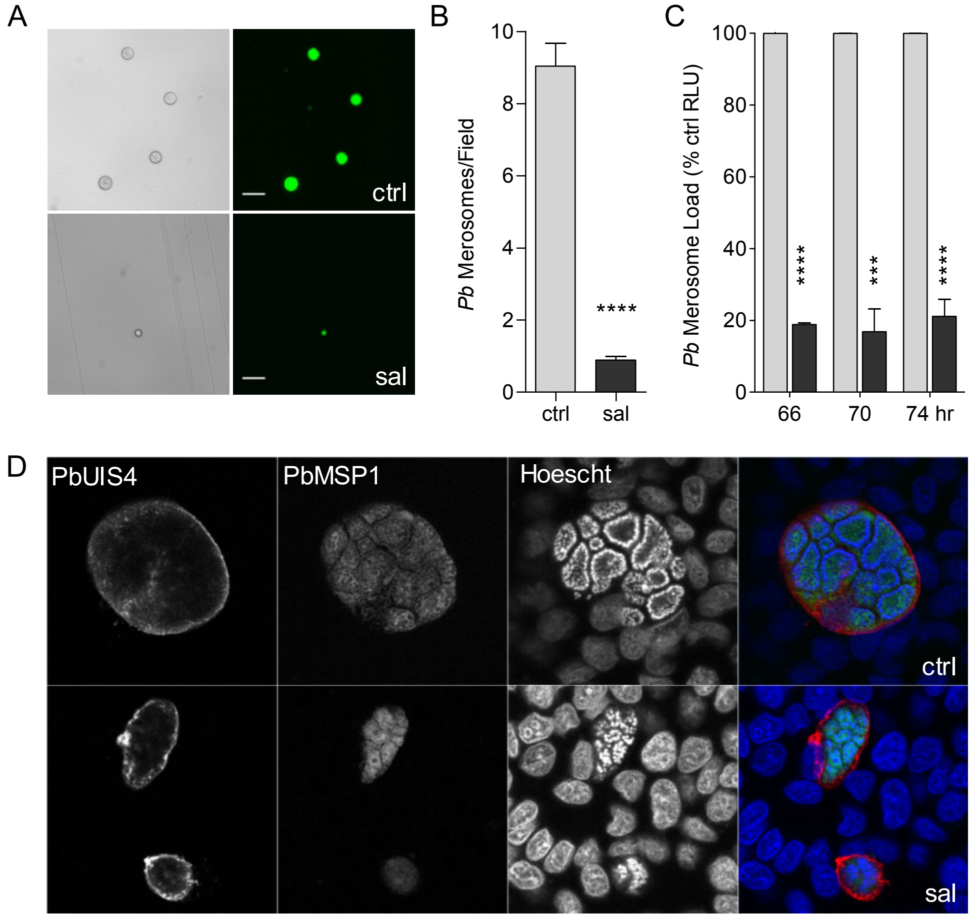

Figure S4. Related to Figure 3. Merosome analysis.
(A-B) Live GFP and bright field representative images (A) and quantification (B) of *P. berghei* detached merosomes at 66 hr after infection of HepG2 cells and treatment with 2.5mM salicylate (sal) or the vehicle control (ctrl, water). Similar results were observed upon treatment with A769662 (*data not shown*). Scale bars, 20μm. ****p<0.0001.
(C) Luminescence levels from detached merosomes at multiple time-points after infection of HepG2 cells with luciferase-expressing *P. berghei* parasites under salicylate treatment (2.5mM). Relative luminescence values (RLU) were measured at 66, 70, and 74 hr. The bars are mean±SEM normalized to the correspondent untreated control, from 3 independent experiments. ***p<0.001; ****p<0.0001.
(D) HepG2 cells at 66 hr after infection, treated with vehicle (ctrl) or 2.5mM salicylate (sal), probing with anti-PbUIS4 (red) and anti-MSP1 (green) antibodies. DNA is stained with Hoechst (blue). Images acquired on a confocal microscope with a 40x magnification.

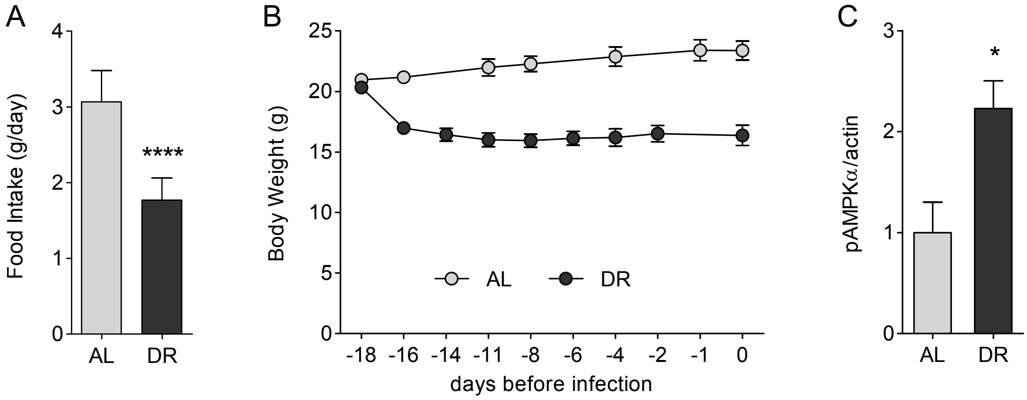

Figure S5. Related to Figure 4. Average food intake (A) and body weight change (B) in male C57BL/6 mice fed *ad libitum* (AL) or in mice under dietary restriction (DR) regimen. Mice on DR were given daily 60-70% of the food consumed by the AL group, for 2 to 3 weeks prior to infection to avoid stress effects and allow for weight stabilization. To determine whether DR was working as expected we monitored body weight every 2-3 days prior to infection. DR mice show approximately 20% loss of the initial body weight. Values are mean±SD (5 mice/group). Representative experiment of 3 independent DR experiments. (C) Quantification of pAMPKα^{T172} in AL and DR mice. Values in the bars are mean±SEM (3 mice/group). *p<0.05; ****p<0.0001.

Table S1. Related to Figure 3. AMPK agonists used in the study.

Compound	Mechanism of AMPK activation	References	*Pb* EC_{50} (μM)
Salicylate	Direct binding to AMPKβ1 subunit	(Hawley et al., 2012)	950±11
A769662	Direct binding to AMPKβ1 subunit	(Goransson et al., 2007)	214±29
Metformin	Direct inhibition of complex 1 of the respiratory chain	(Owen et al., 2000)	156±70
2-Deoxy-D-glucose	Inhibition of glycolysis	(Woodward and Hudson, 1954)	273±53

SUPPLEMENTAL EXPERIMENTAL PROCEDURES

Chemicals

Salicylate (71945), metformin (D150959), and 2-Deoxy-D-glucose (D6134), were obtained from Sigma. A769662 (171258) was purchased from Calbiochem. The stock solutions were as follows: 1M salicylate (dH_2O); 50mM metformin (dH_2O); 100mM 2-Deoxy-D-glucose (dH_2O); 100mM A769662 (DMSO).

Parasite Lines

P. berghei ANKA expressing GFP (259cl2), RFP (733cl1), and Luciferase (676m1cl1) parasite lines were obtained from the Leiden Malaria Research Group (www.pberghei.eu). *P. yoelii* 17X NL parasites were obtained through the MR4 (www.mr4.org). *P. berghei* and *P. yoelii* sporozoites were isolated from salivary glands of *Anopheles stephensi*, bred at Instituto de Medicina Molecular (Lisboa, Portugal). *P. falciparum* sporozoites were obtained by dissection of salivary glands from infected *Anopheles gambiae* mosquitoes obtained from the insectary at Johns Hopkins School of Public Health (Baltimore, USA) or Sanaria Inc. (USA).

Cells Lines and Infections

Cells were cultured in medium supplemented with Fetal Bovine Serum (FBS), 50 µg/mL Penicillin/Streptomycin, and 2mM Glutamine (all Gibco) at 37°C with 5% CO_2. RPMI medium was also supplemented with 0.1mM non-essential amino acids. Cells were always infected 24 hr post seeding. At time of infection, medium was removed and freshly dissected *P. berghei* or *P. yoelii* sporozoites in supplemented medium containing Fungizone (1µg/mL, Gibco) were added to the wells, followed by a 5-minute centrifugation at 3000 rpm. In all assays, unless stated otherwise, medium was changed 2 hr after infection either to add fresh, non-treated medium, or to add different compounds, always supplemented with Fungizone (Gibco). Details of cell lines, numbers of cells seeded, and sporozoites used for different experiments can be found in the table below.

Micropatterned coculture (MPCC) preparation and *P. falciparum* infection were carried out as described previously (Khetani and Bhatia, 2008; March et al., 2013). Briefly, glass-bottom 96-well plates were coated homogenously with rat tail type I collagen (50 µg/ml) and subjected to soft-lithographic techniques to pattern the collagen into microdomains of 500-µm islands that mediate selective hepatocyte adhesion. To create MPCCs, cryopreserved primary human hepatocytes (Life Technologies) were pelleted by centrifugation at 100 × g for 6 min at 4°C, assessed for viability using trypan blue exclusion, and seeded on collagen-micropatterned plates. Each well contained approximately 1×10^4 hepatocytes organized in colonies of 500 µm in serum-free DMEM with Penicillin/Streptomycin. The cells were washed in complete medium 3 hr post seeding, and the medium was switched to human hepatocyte culture medium. One day after seeding, 75×10^3 freshly dissected *P. falciparum* sporozoites were added to each well. The cells were washed twice 3 hr after sporozoite addition, and 7×10^3 3T3-J2 murine embryonic fibroblasts per well were seeded in human hepatocyte culture medium containing 2 mM salicylate or control vehicle. 5 days post infection, cells were fixed and analyzed by immunofluorescence assay.

Mouse Primary Hepatocytes

Primary hepatocytes were isolated from livers of adult C57BL/6 male mice following an adaptation of the previously described two-step *in situ* perfusion method (Liehl et al., 2014; Seglen, 1976). Briefly, mice were sacrificed by CO_2 inhalation and immediately opened to begin the process of perfusion. The portal vein was cannulated with a 26 g needle and liver was perfused with 30-40 mL of Liver Perfusion Medium (LPM, Gibco) at 37°C followed by digestion with 30-40 mL of Liver Digest Medium (LDM, Gibco) at a flow rate of 7-9 mL/min, controlled by a peristaltic pump. Outflow drain was achieved by cutting the inferior vena cava. Following perfusion and digestion, the liver was carefully removed and placed on a cell culture dish containing 10 mL of LDM and the capsule membrane was carefully peeled away with fine tweezers. The liver was gently shaken to release any loose cells. The cell suspension, containing primary hepatocytes, non-parenchymal cells (NPCs), and dead cells, was serially passed through a 100 µm strainer followed by a 70 µm strainer and was washed twice with 30 mL of 4% FBS supplemented William's E Medium (Gibco) at 30 g for 3 minutes at 20°C. The hepatocyte fraction was then purified to remove any contaminating NPCs and dead cells by layering over a 60% Percoll gradient (GE Healthcare) followed by centrifugation for 20 minutes, 750 g at 20°C with no break. Purified hepatocyte pellet was washed twice with William's E Medium, cells were counted and assessed for viability with Trypan blue. Viable hepatocytes were plated on collagen-coated plates and allowed to settle and attach overnight and infected as described above for hepatoma cells.

Cells	Medium	FBS	Plate	Cells/well	Spz	Parasite	Assay
Huh7	RPMI	10%	96	1×10^4	8×10^3	Luciferase	Luminescence
			24	5×10^4	5×10^4	GFP	Microscopy
			24	5×10^4	5×10^4	Luciferase	Microscopy
			24	5×10^4	75×10^3	GFP	Transfection
			24	3×10^4	5×10^4	GFP	siPools KD
		20%	24	8×10^4	7×10^4	GFP/RFP	Sorting
HepG2	DMEM	10%	24	5×10^4	5×10^4	Luciferase	Luminescence
MEFs wt	DMEM	10%	24	3×10^4	5×10^4	GFP	Microscopy
MEFs AMPKα-null	DMEM	10%	24	4×10^4	5×10^4	GFP	Microscopy
Mouse primary hepatocytes	William's E	4%	24	12×10^4	5×10^4	GFP	Microscopy
Hepa1-6	DMEM	10%	24	4×10^4	$3\text{-}4\times10^4$	GFP	Microscopy

Cell Sorting

Huh7 cells in 24-well plates were infected with sporozoites as described in the table above. Cells were harvested at 2 hr after infection by trypsinization, passed through a 70 µm filter, washed with RPMI supplemented with 20% FBS and centrifuged 1200 rpm at room temperature for 5 minutes. Cell pellet was resuspended in RPMI 20% FBS and sorted with BD FACSARIA III sorter (BD Biosciences). Cells were sorted with a gate on the GFP/RFP+ cells (infected) and a gate on the GFP/RFP- population (non-infected), at the same flow rate, and collected into 1 mL of RPMI 20% FBS. Collected samples were kept at 4°C and seeded at a 1:1 ratio (infected:non-infected) onto collagen-coated 48-well plates (5×10^4 cells/well) in RPMI 20% FBS and Fungizone. Cells were harvested at 8 hr, 18 hr, or 30 hr post infection and processed for Western blot as described below.

Western Blotting

Prior to cell lysis, medium was removed and cells were washed with ice-cold PBS. 80 µl of lysis buffer were immediately added and incubated on ice for 15 min. Cells were lysed in ice-cold RIPA buffer (150 mM Sodium Chloride, 1% triton X-100, 0.5% Sodium Deoxycholate, 0.1% SDS and 50 mM Tris pH 8.0) containing protease and phosphatase inhibitors (Complete and phosSTOP, Roche). Cells were harvested with a cell scraper and centrifuged at 14 000 rpm, 4°C for 10 minutes to pellet non-soluble cell material. The supernatant containing soluble fraction was collected and kept at -80°C or processed immediately.

Livers were homogenized in ice-cold lysis buffer (50 mM Hepes, 150 mM NaCl, 10 mM NaF, 1 mM Sodium pyrophosphate, 0.5 mM EDTA, 1 mM DTT, 1% triton, 1 mM Na_3VO_4, 250 mM Sucrose, protease inhibitor cocktail and phosphatase inhibitors).

Total protein content was measured by Bradford Assay (Biorad), according to manufacturer's instructions. 15 µg of total cell or 50 µg of total liver lysates were resolved on either 8% SDS-PAGE or Any kD mini-protean precast gels (Biorad) and transferred to a nitrocellulose membrane using standard wet transfer with 1x Tris-Glycine buffer containing 20% methanol for 2 hr at 100 V constant or were transferred using iBlot gel Transfer stacks (ThermoFisher). Membranes were blocked in TBS-5% BSA Tween 0.2% for 1 hr at room temperature and incubated with primary antibodies overnight at 4°C.

pACC and pAMPK were detected using rabbit anti-phosho-ACC^{S79} (mAb D7D11, 1:1000) and rabbit anti-phospho-$AMPK\alpha^{T172}$ (mAb 40H9, 1:1000), respectively (both from Cell Signaling Technology, CST). Total AMPKα was detected using rabbit antibody (CST mAb D63G4, 1:1000). Anti-GST rabbit polyclonal antibody (Abcam ab9085, 1:500) was used to detect Glutathione S-Transferase. Anti-actin (Sigma-Aldrich A2066, 1:1000) rabbit antibody was used as loading control (incubation for 1 hr at room temperature). Horseradish peroxidase (HRP)-conjugated goat anti-rabbit IgG, Fc fragment specific and HRP-conjugated goat anti-mouse IgG, light chain specific (both from Jackson ImmunoResearch) were used as secondary antibodies.

Luminescence Assay

Infected cells were washed with PBS at 48 hr after infection and lysed in 75 µl of 1x Firefly lysis buffer from Firefly Luciferase Assay Kit (Biotium). Plates were shaken for 20 minutes and then centrifuged for 5 minutes, 3000 rpm. 50 µl of D-Luciferin dissolved in the kit buffer were added to 30 µl of total lysate in white 96-well plates and luciferase activity was measured using a multiplate reader (Infinite 200M, Tecan). Cell viability was assayed by Alamar Blue assay (Invitrogen) using the manufacturer's protocol, following an incubation of 90 minutes at 37°C.

For quantification of detached merosomes, medium was replenished at 48 hr post infection with 50 µl of complete medium. At 66-74 hr, supernatants were collected and lysed with 12.5 µl of 5x Firefly lysis buffer and incubated at room temperature for 10 minutes with gentle shaking. The whole lysate (60 µl) was transferred to a white plate as mentioned above, and luciferase activity was measured after adding 100 µl (1 mg/mL) of D-luciferin dissolved in firefly luciferase assay buffer.

Live Fluorescence Imaging and Quantification of Detached Merosomes

Supernatant (~500 µl) containing detached merosomes from HepG2 cells infected with *P. berghei* expressing GFP (at 66 hr post infection) were collected. Merosomes were pelleted at 1200 rpm for 5 minutes, supernatant removed, and pellet was resuspended in 20 µl of complete DMEM. Merosomes were quantified by loading 10 µl onto a hemocytometer and visualized with an upright widefield fluorescence microscope as described below. At least 10 fields were imaged and quantified per condition. The acquired images were then analyzed on ImageJ (http://imagej.nih.gov/ij/) to quantify the size of the parasite, which is the area of the parasite as determined by fluorescence intensity. Counts were determined as number of merosomes counted per field at 10x magnification.

Immunofluorescence Assay

Infected cells on coverslips (in triplicate) were fixed in 4% Paraformaldehyde (PFA) for 10 minutes, permeabilized in PBS 0.1% Triton-100 for 10 minutes and blocked in PBS 0.1% Triton-100 and 1% BSA for 30 minutes. Primary antibody staining was performed in blocking solution for 1 hr with the following antibodies: goat anti-PbUIS4 (1:1000), mouse anti-PbHSP70 (2E6, 1:200), rabbit anti-PbMSP1 (1:450), mouse anti-PfHSP70 (clone 4C9, 1:200), rabbit anti-GST (Abcam ab9085, 1:400). Primary antibodies were detected using several AlexaFluor-conjugated antibodies (Molecular Probes/Invitrogen): donkey anti-goat 568 (1:400), donkey anti-mouse 488 or 647 (1:400), donkey anti-rabbit 488 (1:400). Coverslips were mounted in Fluoromount-G (Southern Biotech).

Livers were fixed with 4% PFA for 2 hr and sliced into 50 µm-thick sections using the Vibratome VT 1000 S (Leica). Liver sections were permeabilized and blocked in PBS 1% BSA and 0.3% Triton-100 for 45 minutes, and incubated with goat anti-PbUIS4 (1:1000) for 1 hr. After washing in PBS, liver sections were incubated with donkey anti-goat conjugated to Alexa Fluor 555 (1:400) and Hoechst (1:1000) for another 1 hr and mounted in Fluoromount-G. All incubations were performed at room temperature.

Imaging Analysis

30 fields in each coverslip (triplicates) were randomly acquired using the MetaMorph software (Molecular Devices) with an inverted wide-field fluorescence microscope, Axiovert 200M (Zeiss) with a 20x magnification. For transfected cells and liver sections, acquisition was performed by non-random identification of parasites in the entire coverslip. The acquired images were then analyzed on ImageJ (http://imagej.nih.gov/ij/) to quantify parasite size, determined as the area of parasite defined by the staining of PbUIS4 or HSP70. We established a minimum size cut-off of 10 μm^2. The total number of cells was estimated by the total number of nuclei per image, and used to obtain the percentage of infected cells.

Illustrative images of *P.berghei* schizonts (Figure S4) and infected liver sections (Figure 4) were taken on confocal microscope, LSM 510 Meta (Zeiss) and *P. falciparum* liver schizonts were imaged on Nikon A1R Ultra-Fast Spectral Scanning Confocal Microscope (Figure 3F). Live GFP imaging of merosomes were taken on fluorescence microscope Leica DM5000B (Figure 3H, S4A).

siRNA Knockdown

Knockdown experiments were performed in 24-well plates containing coverslips for microscopy using a reverse transfection protocol. Huh7 cells were trypsinized, resuspended in antibiotic-free complete RPMI, counted, and prepared for seeding at a final concentration of 75×10^3 cells/mL. Meanwhile, siRNA complexes, targeting multiple sites on a single transcript to increase specificity and avoid off-targets (Hannus et al., 2014), were prepared following manufacturer's protocol. Briefly, siPool oligonucleotides (siTools Biotech GmbH, Martinsried, Germany) targeting human *prkaa1a* (NM_006251) and *prkaa2a* (NM_006252), or control siPool were pre-mixed in 100 µl of Opti-MEM (Gibco) with 0.2 µl of Lipofectamine RNAiMax (Invitrogen) transfection reagent for a final concentration of 3 nM siPool/well. Complexes of siPool/RNAiMax were aliquoted (100 µl) into each well and incubated at room temperature for 15 minutes. Cells were then seeded on top of the complexes in a total volume of 400 µl at seeding density of 3×10^4 cells/well. 24 hr post transfection, medium was replenished with complete RMPI containing antibiotics. 48 hr post transfection, cells were harvested for assessment of knockdown efficiency by Western blot and/or infected with 5×10^4 *P. berghei* sporozoites/well for microscopic analysis of parasite infection.

Site Directed Mutagenesis and Transfection

For AMPKα overexpression in cells, the following plasmids were used: empty plasmid (Tanaka 1995; pEBG; 22227, Addgene), pEBG-AMPKα1 (1-312) plasmid (Crute et al., 1998; Egan et al., 2011; 27632, Addgene), and pEBG-AMPKα1 was mutated by site-directed mutagenesis to make T172A. Site-directed mutagenesis was performed with the QuickChange II XL Site-Directed Mutagenesis Kit (Agilent Technologies) according to manufacturer's instructions and verified by sequencing. Primer pairs are as follows: GGTGAATTTTTAAGAGCGAGCTGTGGCTCGCCCAATTATGCTGC, and GCAGCATAATTGGGCGAGCCACAGCTCGCTCTTAAAAATTCACC.

Cells were reverse transfected with the AMPKα CA, AMPKα T172A and empty plasmids, using FuGENE 6 Transfection Reagent (Promega). DNA was added to the transfection reagent in a ratio of 3:1, to a final amount of 0.5 µg of DNA/well. Cells were infected or lysed (for Western blot) 24 hr after transfection.

Flow Cytometry

The percentage of infected red blood cells (parasitemia) at 72 hr after injection of GFP-expressing sporozoites into mice was determined by flow cytometry (LSR Fortessa, BD Biosciences). A drop of blood was collected from the mouse tail into ~500 µl PBS, and 1-2×10^6 cells per mouse were analyzed (medium flow speed). Cells were selected on the basis of their size by gating first on FSC and SSC and, subsequently, on FITC (green) and PE (red) channels. The GFP-expressing parasites were detected in the FITC channel. False GFP positive cells (red blood cell's auto-fluorescence) were eliminated by plotting FITC against PE. The analysis was performed by using the FlowJo software (TreeStar, USA).

SUPPLEMENTAL REFERENCES

Crute, B.E., Seefeld, K., Gamble, J., Kemp, B.E., and Witters, L.A. (1998). Functional domains of the alpha1 catalytic subunit of the AMP-activated protein kinase. The Journal of biological chemistry *273*, 35347-35354.

Egan, D., Kim, J., Shaw, R.J., and Guan, K.L. (2011). The autophagy initiating kinase ULK1 is regulated via opposing phosphorylation by AMPK and mTOR. Autophagy *7*, 643-644.

Goransson, O., McBride, A., Hawley, S.A., Ross, F.A., Shpiro, N., Foretz, M., Viollet, B., Hardie, D.G., and Sakamoto, K. (2007). Mechanism of action of A-769662, a valuable tool for activation of AMP-activated protein kinase. The Journal of biological chemistry *282*, 32549-32560.

Hannus, M., Beitzinger, M., Engelmann, J.C., Weickert, M.T., Spang, R., Hannus, S., and Meister, G. (2014). siPools: highly complex but accurately defined siRNA pools eliminate off-target effects. Nucleic acids research *42*, 8049-8061.

Hawley, S.A., Fullerton, M.D., Ross, F.A., Schertzer, J.D., Chevtzoff, C., Walker, K.J., Peggie, M.W., Zibrova, D., Green, K.A., Mustard, K.J.*, et al.* (2012). The ancient drug salicylate directly activates AMP-activated protein kinase. Science *336*, 918-922.

Khetani, S.R., and Bhatia, S.N. (2008). Microscale culture of human liver cells for drug development. Nature biotechnology *26*, 120-126.

Liehl, P., Zuzarte-Luis, V., Chan, J., Zillinger, T., Baptista, F., Carapau, D., Konert, M., Hanson, K.K., Carret, C., Lassnig, C.*, et al.* (2014). Host-cell sensors for Plasmodium activate innate immunity against liver-stage infection. Nature medicine *20*, 47-53.

March, S., Ng, S., Velmurugan, S., Galstian, A., Shan, J., Logan, D.J., Carpenter, A.E., Thomas, D., Sim, B.K., Mota, M.M.*, et al.* (2013). A microscale human liver platform that supports the hepatic stages of Plasmodium falciparum and vivax. Cell host & microbe *14*, 104-115.

Owen, M.R., Doran, E., and Halestrap, A.P. (2000). Evidence that metformin exerts its anti-diabetic effects through inhibition of complex 1 of the mitochondrial respiratory chain. The Biochemical journal *348 Pt 3*, 607-614.

Seglen, P.O. (1976). Preparation of isolated rat liver cells. Methods in cell biology *13*, 29-83.

Woodward, G.E., and Hudson, M.T. (1954). The effect of 2-desoxy-D-glucose on glycolysis and respiration of tumor and normal tissues. Cancer research *14*, 599-605.
