# Supplementary material for: Host AMPK Is a Modulator of Plasmodium Liver Infection
Source: Cell Rep. 2016 Aug 25;16(10):2539–45. doi: 10.1016/j.celrep.2016.08.001 (PMC5014760; doi:10.1016/j.celrep.2016.08.001)
Supplement: Document S2. Article plus Supplemental Information [file mmc2.pdf]

Cell Reports

Report

Host AMPK Is a Modulator of *Plasmodium* Liver Infection

Graphical Abstract

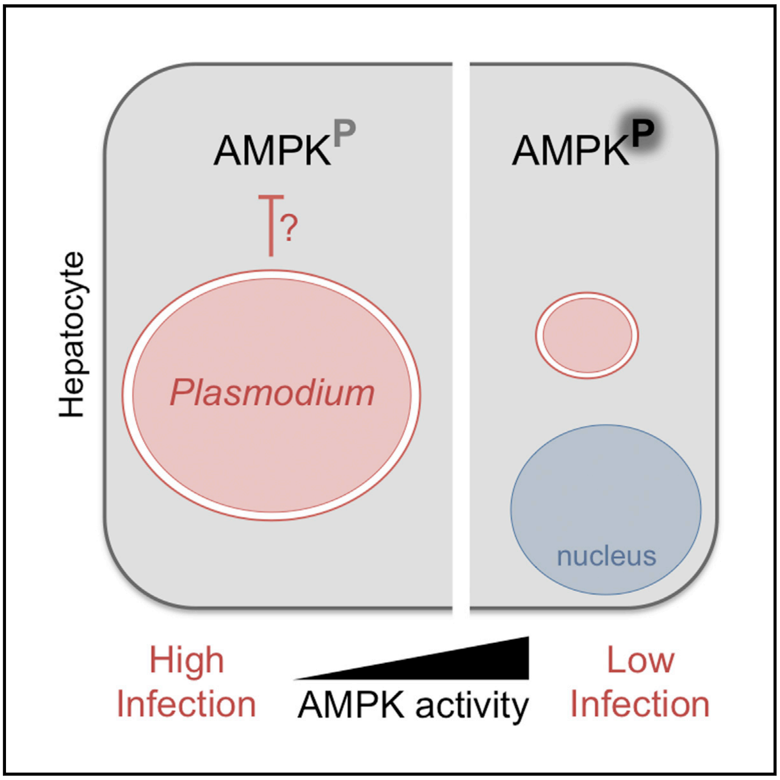

Highlights

- *Plasmodium*-infected hepatic cells exhibit decreased AMPK activity
- AMPK suppression favors hepatic infection; its activation reduces parasite development
- AMPK activating compounds efficiently reduce liver infection in vitro and in vivo

Authors

Margarida T. Grilo Ruivo, Iset Medina Vera, Joana Sales-Dias, ..., Sangeeta N. Bhatia, Maria M. Mota, Liliana Mancio-Silva

Correspondence

mmota@medicina.ulisboa.pt (M.M.M.), lilianamancio@medicina.ulisboa.pt (L.M.-S.)

In Brief

AMPK is a stress-activated kinase that regulates cellular energy homeostasis. Ruivo et al. show that AMPK signaling is relevant to hepatocyte infection by malaria parasites. Induction of host AMPK activity affects the ability of the host cell to support parasite growth in the liver, thus reducing the subsequent malaria burden.

Ruivo et al., 2016, Cell Reports *16*, 2539–2545
September 6, 2016 © 2016 The Authors.
http://dx.doi.org/10.1016/j.celrep.2016.08.001

Cell Reports
Report

OPEN ACCESS
CellPress

Host AMPK Is a Modulator of *Plasmodium* Liver Infection

Margarida T. Grilo Ruivo,[1,3] Iset Medina Vera,[1,3] Joana Sales-Dias,[1] Patrícia Meireles,[1] Nil Gural,[2] Sangeeta N. Bhatia,[2] Maria M. Mota,[1,4,*] and Liliana Mancio-Silva[1,*]
[1]Instituto de Medicina Molecular, Faculdade de Medicina, Universidade de Lisboa, 1649-028 Lisboa, Portugal
[2]Department of Health Sciences and Technology, Massachusetts Institute of Technology, Cambridge, MA 02142, USA
[3]Co-first author
[4]Lead Contact
*Correspondence: mmota@medicina.ulisboa.pt (M.M.M.), lilianamancio@medicina.ulisboa.pt (L.M.-S.)
http://dx.doi.org/10.1016/j.celrep.2016.08.001

SUMMARY

Manipulation of the master regulator of energy homeostasis AMP-activated protein kinase (AMPK) activity is a strategy used by many intracellular pathogens for successful replication. Infection by most pathogens leads to an activation of host AMPK activity due to the energetic demands placed on the infected cell. Here, we demonstrate that the opposite is observed in cells infected with rodent malaria parasites. Indeed, AMPK activity upon the infection of hepatic cells is suppressed and dispensable for successful infection. By contrast, an overactive AMPK is deleterious to intracellular growth and replication of different *Plasmodium* spp., including the human malaria parasite, *P. falciparum*. The negative impact of host AMPK activity on infection was further confirmed in mice under conditions that activate its function. Overall, this work establishes the role of host AMPK signaling as a suppressive pathway of *Plasmodium* hepatic infection and as a potential target for host-based antimalarial interventions.

INTRODUCTION

Plasmodium spp. are obligate intracellular protozoan parasites and the etiological agents of malaria, an infectious disease that causes major morbidity and mortality and cripples socioeconomic growth. Lack of an effective vaccine and resistance to treatments are setbacks for controlling the disease (World Health Organization, 2015). Malaria infection begins in the liver, when the transmissive forms (sporozoites) invade and replicate by schizogony into thousands of new parasites (merozoites) inside hepatocytes. This high replicative capacity occurs within 48 hr in rodent parasites and up to 2 weeks in human parasites. Despite clear parasitism and subversion of host cell resources during hepatic infection, little is known about how *Plasmodium* infection modifies hepatocyte signaling. Previous transcriptional and post-transcriptional studies provide evidence of parasite-mediated alterations to host cell processes (Albuquerque et al., 2009; Kaushansky et al., 2013). Nonetheless, a comprehensive understanding of the hepatocyte response to this first stage of *Plasmodium* infection is needed to devise new antimalarial interventions.

Many intracellular pathogens actively alter host cellular metabolism as a strategy to produce optimal conditions for proliferation. An obvious metabolic target is AMPK (AMP-activated protein kinase), the master regulator of cellular energy homeostasis. AMPK is a conserved heterotrimeric (α catalytic, β and γ regulatory subunits) serine/threonine kinase that, as its name implies, responds to an increased AMP/ATP ratio. AMPK activation influences diverse pathways from glucose and lipid metabolism to cell-cycle regulation, promoting catabolism and inhibiting ATP consuming processes (reviewed in Hardie, 2014).

Manipulation of host AMPK activity is described for virus, bacteria, and parasite infections. For example, *Mycobacterium*, *Leishmania*, human cytomegalovirus, vaccinia, and simian vacuolating virus 40 induce AMPK activity, while hepatitis C virus (HCV) suppresses AMPK activity (Moreira et al., 2015; Singhal et al., 2014; reviewed in Brunton et al., 2013). Thus, AMPK modulation varies with the pathogen and the host cellular context and is dependent on the specific energetic requirements.

In this study, we investigated the role of host AMPK during the course of *Plasmodium* hepatic infection. We show that host AMPK function is suppressed during infection by these parasites. Using several in vitro and in vivo approaches, we demonstrate that activation of the AMPK signaling pathway impairs the intracellular replication of malaria liver-stage parasites.

RESULTS

Plasmodium Hepatic Infection Leads to Decreased AMPK Function

AMPK activity can be determined by the phosphorylation of a threonine (T172) residue in the AMPKα catalytic subunit as well as the phosphorylation of the main downstream effector acetyl-coA (coenzyme A) carboxylase (ACC, S79), a rate-limiting enzyme in fatty acid synthesis (Hardie and Pan, 2002). To test

This is an open access article under the CC BY license (http://creativecommons.org/licenses/by/4.0/).

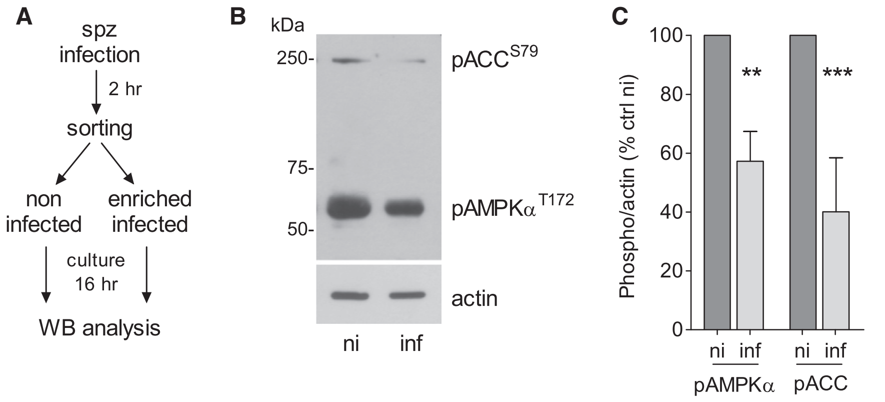

Figure 1. *P. berghei* Hepatic Infection Alters the AMPK Activation Status

(A) Timeline of infection and sample collection. Huh7 cells were infected with GFP-expressing *P. berghei* sporozoites (spz) and subjected to fluorescence-activated cell sorting to separate infected from non-infected (ni) cells at 2 hr post-infection. Cells were re-plated 1:1 (infected:non-infected), cultured for 16 hr, and compared to non-infected by western blot (WB).

(B and C) WB analysis of lysates from non-infected (ni) and enriched infected (inf) Huh7 cells collected at 18 hr post-infection, probing with anti-phospho-AMPKα (pAMPKα^{T172}), -phospho-ACC (pACC^{S79}), and -actin antibodies. (B) Representative blot and (C) quantitative analysis (mean ± SEM) of three independent experiments. Analysis of additional time points and control (ctrl) for total AMPKα abundance is shown in Figure S1. $^{**}p < 0.01$; $^{***}p < 0.001$.

whether AMPK activation is altered upon *Plasmodium* infection, we compared the phosphorylation status of AMPKα and ACC in non-infected Huh7 cells versus cells infected with the rodent parasite *P. berghei* (Figure 1A). Phosphorylation of AMPKα and ACC is lower in infected cells when compared to the non-infected cells at 18 hr post-infection ($p < 0.01$; Figures 1B and 1C). We confirmed a decrease in AMPKα phosphorylation over time (Figure S1A) and verified that total AMPKα abundance is not altered during infection (Figure S1B). A general reduction in phosphorylation was ruled out, since we observed a modest increase in phospho-Akt levels, as previously reported (data not shown; Kaushansky et al., 2013).

Modulation of Host AMPK Affects *P. berghei* Hepatic Development In Vitro

Next, we investigated whether AMPK function could impact *P. berghei* infection. AMPKα catalytic subunit is encoded by two distinct genes, *prkaa1* (AMPKα1) and *prkaa2* (AMPKα2), which are expressed in hepatocytes. We knocked down both subunits by RNAi 48 hr prior to infection and confirmed a decrease in AMPKα and ACC phosphorylation at the time of infection (Figures 2A and 2B). Microscopic analysis of *P. berghei*-infected Huh7 cells at 48 hr post-infection revealed a small, but significant, increase in mean size distribution of schizont parasite forms (194 ± 127 μm^2 versus 150.9 ± 99 μm^2, $p < 0.0001$; Figure 2C). We confirmed this difference in parasite size by testing infection in mouse embryonic fibroblasts (MEFs) lacking both catalytic subunits (Laderoute et al., 2006) (291.2 ± 175 μm^2 versus 176.8 ± 116 μm^2, $p < 0.0001$; Figure 2D).

To test whether AMPK function might hinder infection, we overexpressed a constitutively active (CA) form of AMPKα1 subunit (Crute et al., 1998) in Huh7 cells. As controls, we expressed an inactive mutant AMPKα1 variant (T172A) and an empty plasmid (Figures 2E and S2A). AMPKα and ACC phosphorylation status was monitored by western blot analysis (Figure 2F). Microscopy examination at 48 hr post-infection revealed no significant difference in parasite size in cells not expressing the plasmids (Figure S2B). However, cells expressing the CA plasmid harbored significantly smaller hepatic schizonts, compared to controls (CA, 132.9 ± 83 μm^2; T172A, 207.2 ± 119 μm^2; and empty, 198.4 ± 92 μm^2, $p < 0.01$; Figures 2G and 2H), implying that increased host AMPK activity decreases *P. berghei* hepatic growth.

AMPK Agonists Restrict *Plasmodium* Hepatic Infection In Vitro

The impact of host AMPK activation during *P. berghei* infection was further characterized using a pharmacological approach. We exposed infected cells to known AMPK-activating compounds (salicylate, metformin, 2-deoxy-D-glucose, and A769662) (Hardie, 2014) (Table S1) and analyzed infection via luminescence and immunofluorescence assays in Huh7 cells (Figure S3). A dose-dependent reduction of total parasite load was observed for all tested compounds, with calculated half maximal effective concentration (EC_{50}) values ranging from 200 μM to 1 mM (Figure S3A; Table S1), which are within or below the range described for other mammalian cell systems. Microscopy analysis revealed that AMPK-activating compounds led primarily to a significant decrease in schizont size, but not parasite numbers (Figures S3B and S3C).

To dissect the effect of host AMPK activation on parasite infection, we focused on salicylate, known to bind the AMPKβ1 subunit promoting AMPKα T172 phosphorylation (Hawley et al., 2012) (Figures 3A and 3B). The data show a similar negative effect on parasite development in Huh7 cells (40 ± 20.3 μm^2 versus 177 ± 101.5 μm^2, $p < 0.0001$; Figure 3C) and mouse primary hepatocytes infected with *P. berghei* (94.36 ± 36 μm^2 versus 272.2 ± 209 μm^2, $p < 0.0001$; Figure 3D), Hepa1-6 cells infected with *P. yoelii* (71 ± 42 μm^2 versus 180.9 ± 106 μm^2, $p < 0.0001$; Figure 3E), and human primary hepatocytes derived from different donors infected with *P. falciparum* (38 ± 23.9 μm^2 versus 84 ± 40.9 μm^2, $p < 0.0001$; Figure 3F). Thus, treatment with salicylate during hepatic infection leads to a reduction in parasite size, regardless of host cell or *Plasmodium* species.

To determine the time-course kinetics during which activated AMPK restricts parasite development, we exposed cells to salicylate at different time intervals post-infection. We observed that the parasite is most susceptible to salicylate treatment

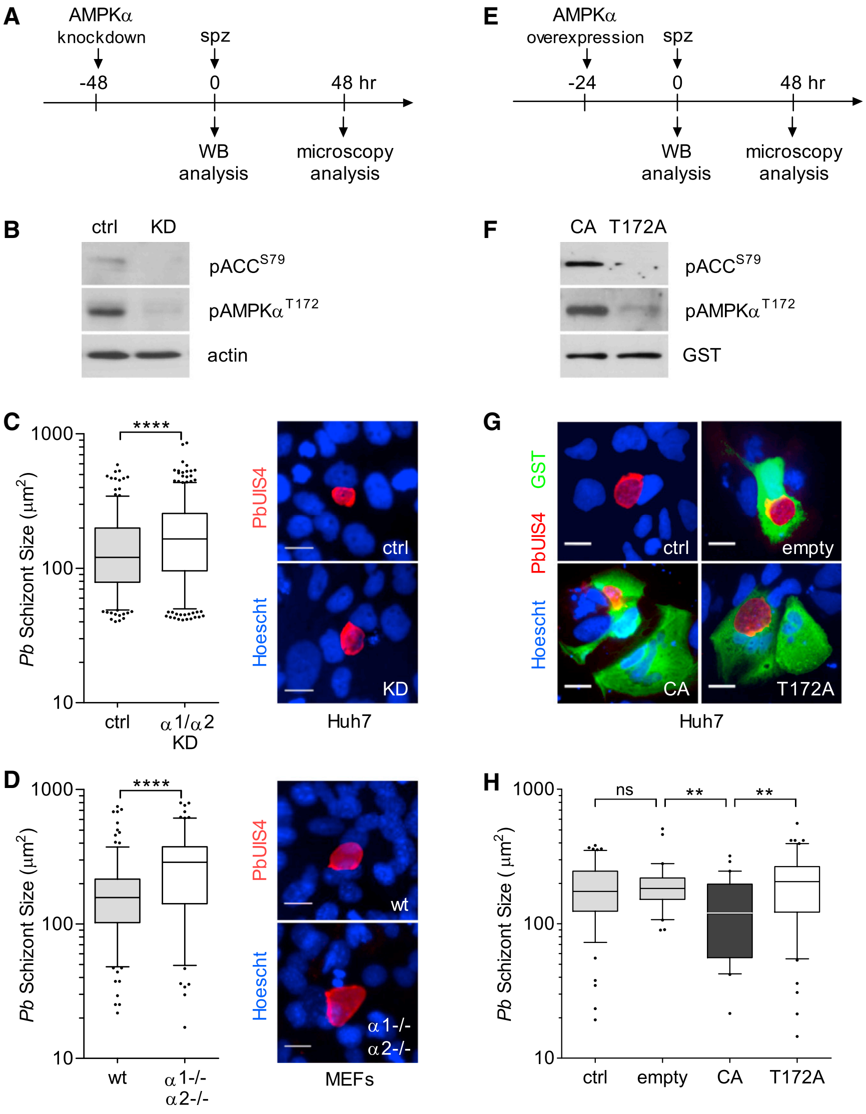

Figure 2. Modulation of Host AMPK Activity Alters *P. berghei* Development

(A) Timeline of RNAi knockdown (KD) and infection.

(B) pAMPKα^{T172} and pACC^{S79} status in lysates of Huh7 cells 48 hr after AMPK α1 and α2 KD. Representative blot of three independent experiments (KD efficiency, mean±SEM, 64.3% ± 10.3%).

(C and D) Quantification of parasite size in AMPKα1/α2-depleted Huh7 cells (C) or AMPKα1$^{-/-}$α2$^{-/-}$ MEFs (D), assessed by microscopy at 48 hr post-infection. Parasite size is the area defined by staining with the parasite membrane marker PbUIS4, as shown in the representative images. Nuclei were stained with Hoechst. More than 100 parasites were imaged and analyzed for each of the three independent experiments. ctrl, control; wild type (WT). Scale bars, 20 μm. ****$p < 0.0001$. The outliers in the boxplots represent 5% of data points.

(E) Timeline of transfection with AMPKα1-carrying plasmids and infection.

(F) Representative western blot of pAMPKα^{T172} and pACC^{S79} in lysates of Huh7 cells transfected with the truncated AMPKα1, constitutively active (CA), and mutated AMPKα1 (T172A) plasmids (see schematic of AMPKα1 domains and GST-tagged constructs in Figure S2A). GST was probed to detect transgenes.

(G and H) Representative images (G) and quantification (H) of parasite size in cells expressing AMPKα1-CA, AMPKα1-T172A, or GST only (empty plasmid) in transfected or untransfected cells (ctrl). Transfected cells were identified with anti-GST antibodies, and parasites were detected with anti-PbUIS4. Nuclei were stained with Hoechst. Parasite size distribution in GST-negative cells is shown in Figure S2B. A representative of three independent experiments is shown (30–60 parasites examined per condition). The outliers in the boxplot represent 10% of data points. Scale bars, 20 μm. **$p < 0.01$; ns, non-significant.

during the first 24 hr (Figure 3G). We then allowed *P. berghei* to fully mature in vitro under salicylate treatment into the final end-stage of hepatic development, when merosomes containing fully mature merozoites are released from the substratum (66 hr; Sturm et al., 2006). First, we visualized the live GFP signal of detached merosomes from GFP-expressing *P. berghei*-infected cells at 66 hr and observed a reduction in merosome size (239 ± 135 μm^2 versus 411 ± 311 μm^2, $p < 0.01$; Figures 3H and S4A) and numbers (0.9 ± 0.9 per field versus 9 ± 4.5 per field, $p < 0.0001$; Figure S4B). Then, we examined luminescence levels from luciferase-expressing detached merosomes and observed an 80% reduction in total load up to 74 hr ($p < 0.0001$; Figure S4C), indicating that the decrease was not simply a delay in merosome release. Additionally, we performed immunofluorescence analysis with the merozoite surface marker (MSP1), essential for merozoite maturation, and observed that salicylate-treated cells contained smaller MSP1-positive schizonts (Figure S4D). The data demonstrate that AMPK agonists cause a reduction in parasite development during schizogony, with decreased release of merosomes, suggesting that the total number of merozoites reaching the blood to infect erythrocytes would be lower.

AMPK Activation Reduces *P. berghei* Infection in Mice

Next, we asked whether our in vitro findings were relevant to an in vivo setting. First, we injected mice with salicylate to boost AMPK activity (Hawley et al., 2012) and confirmed increased AMPKα phosphorylation in mouse livers (Figures 4A and 4B). Then, mice were infected by intradermal injection of sporozoites, mimicking a natural mosquito bite. Parasite development under salicylate treatment mirrored the effects observed in vitro, with a significant reduction in size compared to control mice at 42 hr of infection (150.2 ± 110 μm^2 versus 501.9 ± 35 μm^2, $p < 0.0001$; Figures 4C and 4D). Next, we used flow cytometry to monitor the number of infected erythrocytes 72 hr after infection and observed a decrease in pre-patent parasitemia by 57% upon

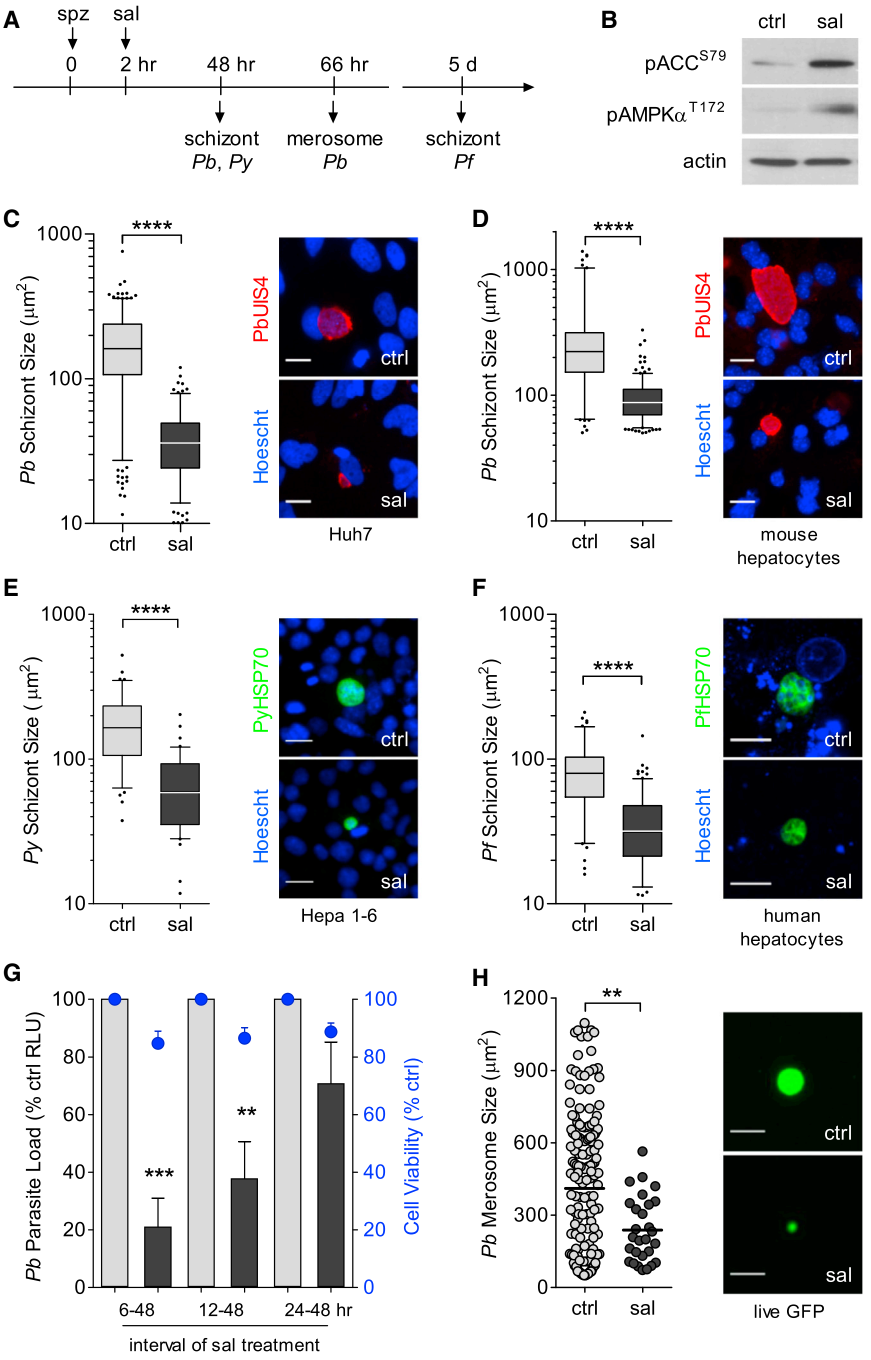

Figure 3. Pharmacological Activation of AMPK Reduces *Plasmodium* Infection

(A) Timeline of infection and microscopy analysis upon treatment with salicylate (sal) or vehicle (ctrl, water) at 2 hr post-infection. Dose-dependent effects of salicylate and other AMPK agonists (metformin, 2-deoxy-D-glucose, and A769662) are shown in Figure S3.

(B) Representative western blot of pAMPKα^{T172} and pACC^{S79} in lysates of non-infected Huh7 cells treated with salicylate (2.5 mM) for 24 hr.

(C–F) Effect of salicylate treatment in Huh7 (C) or mouse primary hepatocytes (D) infected with *P. berghei* (*Pb*), Hepa1–6 cells infected with *P. yoelii* (*Py*) (E), and human primary hepatocytes infected with *P. falciparum* (*Pf*) (F). *Pb* and *Py*, 2.5 mM; *Pf,* 2 mM salicylate. Boxplots of parasite size distribution and illustrative images of three to four independent experiments are shown. Parasite size was determined based on the UIS4 or HSP70 signal after immunofluorescence assays. Nuclei were stained with Hoechst. *Pb* and *Py* scale bars, 20 μm; *Pf* scale bar, 10 μm. ****$p < 0.0001$.

(G) Time-course analysis of salicylate treatment (2.5 mM) starting at 6, 12, and 24 hr after infection of Huh7 cells with luciferase-expressing *P. berghei* parasites. Relative luminescence values (RLU) were measured at 48 hr. The bars are means ± SEM normalized to corresponding control, from three independent experiments. Cell viability (right y axis), measured by Alamar blue, is represented by the blue data points above each bar. **$p < 0.01$; ***$p < 0.001$.

(H) Size distribution scatterplot of detached merosomes from GFP-expressing *P. berghei*-infected HepG2 cells treated with salicylate (2.5 mM) from 2 to 66 hr. Data plotted is mean±SD, vehicle 411±311μm^2, salicylate 239±135μm^2. Data obtained from 3 independent experiments. Live GFP images of representative merosomes are shown. Bright-field images and quantification of detached merosome numbers are in Figures S4A and S4B. Data were obtained from three independent experiments. See Figure S4C for merosome analysis at later time points (66–74 hr) and Figure S4D for MSP1 staining at 66 hr. Scale bars, 50 μm. **$p < 0.01$.

three doses of salicylate ($p < 0.01$; Figure 4E). A single dose was not sufficient to cause a significant reduction in parasitemia (data not shown).

As an alternative, we used a dietary restriction protocol, a method that activates AMPK via alterations in AMP/ATP ratios (Hardie, 2014). We restricted mice food intake by 30%–40% for 2–3 weeks prior and during liver-stage infection, leading to the expected body weight loss (Figures 4F, S5A, and S5B) and efficiently increased liver AMPK activation (Figures 4G and S5C). Physiological activation of AMPK resulted in a significant reduction of hepatic schizont size (252.8 ± 34 μm^2 versus 399.6 ± 29 μm^2, $p < 0.0001$; Figures 4H and 4I) and pre-patent blood stage infection (66% reduction, $p < 0.01$; Figure 4J), similar to salicylate treatment. Altogether, these results show that induction of host AMPK activity affects the ability of the host cell to support parasite growth in the liver, thus reducing the subsequent malaria burden.

DISCUSSION

The present study identifies host cell AMPK signaling as relevant to malaria liver-stage infection. We demonstrate that, while suppression of host AMPK favors *Plasmodium* hepatic

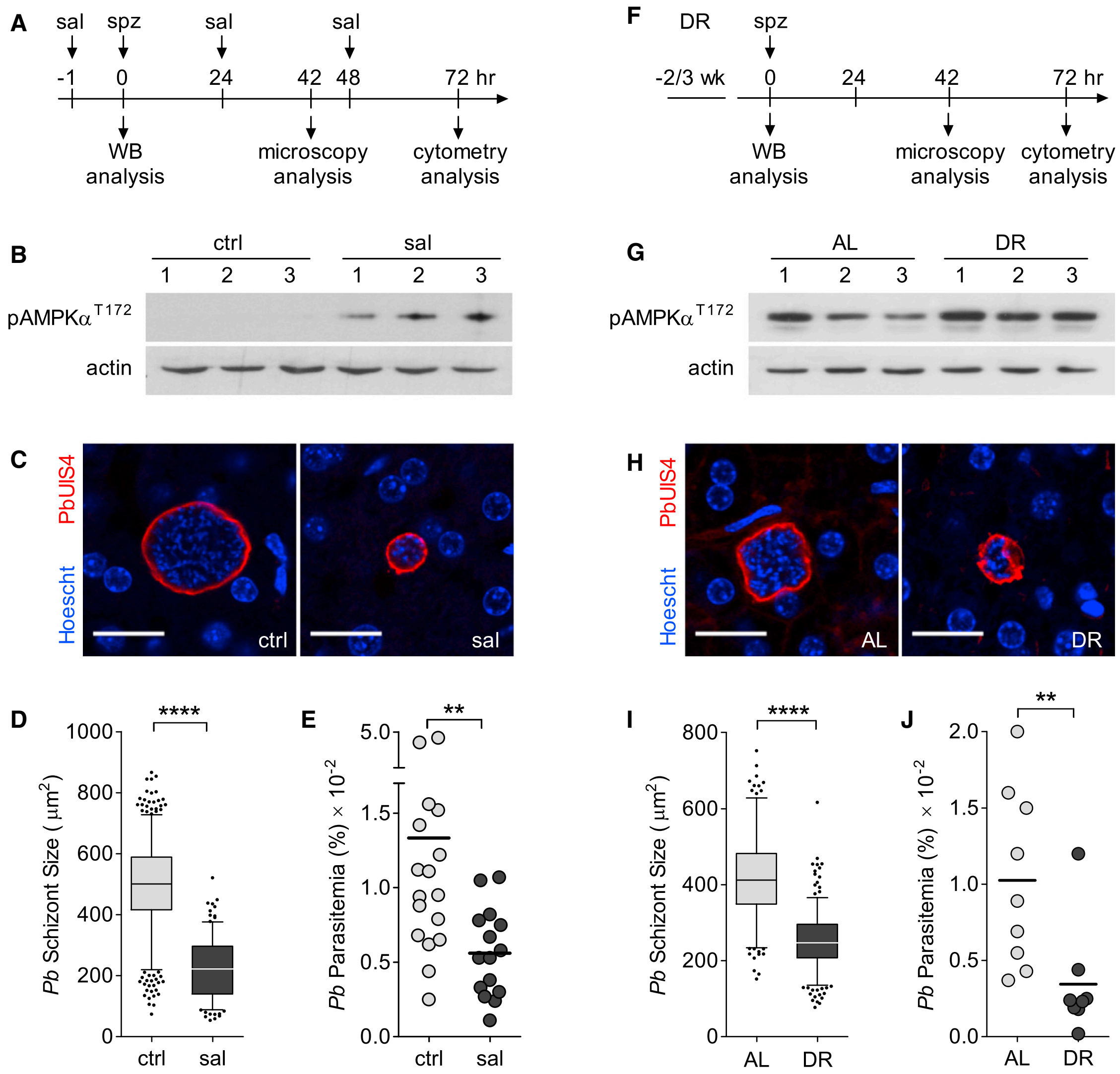

Figure 4. In Vivo Activation of AMPK Reduces Liver-Stage Infection

(A–E) C57BL/6 mice treated with salicylate (sal, 300 mg/kg) or vehicle (ctrl, NaCl 0.9%).

(F–J) C57BL/6 mice fed ad libitum (AL) or a dietary restriction (DR) regimen. Food intake and body weight changes are shown in Figures S5A and S5B.

(A and F) Schedule of the treatments/diets, infections, and sample collection.

(B and G) Western blot of pAMPKα^{T172} status in liver homogenates from non-infected mice 1 hr after injection of salicylate or vehicle (B) and non-infected mice on AL and DR diets (G). Quantification of pAMPKα^{T172} for AL and DR mice is given in Figure S5C. Numbers 1–3 represent individual mice.

(C and H) Confocal representative images from infected livers. Scale bars, 20 μm.

(D and I) Microscopy quantification of *P. berghei* size (area) in liver sections at 42 hr after infection. The parasite area was obtained after immunostaining with anti-PbUIS4 antibodies, as in Figures 2 and 3. Data were pooled from three mice per group (>100 parasites per mouse). The outliers in the boxplots represent 5% of data points.****$p < 0.0001$.

(E and J) Percentage of infected erythrocytes (parasitemia) measured by flow cytometry (≥8 mice per group) at 72 hr after infection. Data plotted is mean±SEM (×10^{-2}), vehicle 1.33±0.28, salicylate 0.56±0.08, AL 1.03±0.19, DR 0.34±0.13. Data were pooled from two independent experiments. **$p < 0.01$.

infection, its activation has a negative impact on parasite growth. The results provide further insights into host hepatocyte signaling and reveal an emerging pattern where the host cell has increased Akt activity, decreased p53 (Kaushansky et al., 2013), and, as shown here, decreased AMPK activity. One advantage of such alterations in the infected cell is a metabolic state that supports rapid proliferation, known as the Warburg effect, a strategy that appears to be used by the parasite itself during schizogony, at least during erythrocytic stages (Salcedo-Sora et al., 2014).

Suppression of AMPK during hepatocyte infection may create a permissive environment serving multiple purposes, for example, through the inhibition of host autophagy (Kim et al., 2011), which may lead to parasite elimination. Alternatively, inhibition of AMPK and downstream targets (e.g., ACC) may help maintain the host cell biosynthetic capacity to sustain

massive parasite replication. Indeed, *Plasmodium* is auxotrophic for certain metabolites, such as cholesterol (Labaied et al., 2011) and lipoic acid (Deschermeier et al., 2012), and scavenges host-derived phosphatidylcholine from hepatocytes (Itoe et al., 2014). A halt in cholesterol and fatty acid synthesis and breakdown, when AMPK is chronically activated, could have a negative impact on parasite growth. Such a mechanism has been described for HCV and Rift Valley Fever virus infections (Mankouri et al., 2010; Moser et al., 2012).

How are the levels of active AMPK lowered and maintained low during infection? This process can be a coping response from the host cell to the invading pathogen or a process prompted by the parasite. *Plasmodium* may actively promote inactivation of AMPK via its own effector molecules or indirectly through modulation of other host cell signaling pathways, leading to decreased AMPK function. As a member of the phylum Apicomplexa, *Plasmodium* sporozoites possess specialized organelles (micronemes and rhoptries) that secrete and inject molecules into host cells during invasion (Kemp et al., 2013). Furthermore, *Plasmodium* is also known to transport proteins beyond the parasite confines during intracellular hepatic growth (Kalanon et al., 2016; Singh et al., 2007). Alternatively, the sporozoite, known to traverse several hepatocytes before final invasion (Mota et al., 2001; Risco-Castillo et al., 2015), may establish infection in a cell with pre-existing low AMPK activity. Whether malaria sporozoites select to home in a cell with suppressed AMPK or modulate host AMPK activity via secretion/transportation of parasite-derived effector molecules remains to be determined.

AMPK activation via small-molecule treatment has been extensively studied, as clinically available drugs (salicylate and metformin) are widely used for treating conditions such as inflammation and diabetes, and are now being evaluated for their anti-tumorigenic properties (Hardie, 2014). Our results demonstrate that salicylate treatment of hepatocytes infected with rodent and human malaria parasites results in reduced parasite replication, which was also shown in vivo with *P. berghei* and is consistent with the effect of overexpressing a constitutively active AMPK in vitro. One caveat of using small molecules to induce AMPK activity is the possible lack of specificity. Salicylate, for example, at high doses has been described to uncouple mitochondria respiration and inhibit necrosis factor κB (NF-κB) signaling (Hawley et al., 2012; Steinberg et al., 2013). Thus, we cannot exclude that our observations with salicylate on parasite replication are fully AMPK dependent. Future experiments using liver-specific genetic mouse models of AMPK are necessary to assess the specificity of salicylate treatment or food restriction effect on liver infection. Furthermore, it would be worthwhile to investigate the impact of AMPK during *Plasmodium* infection of erythrocytes, where AMPK is important to regulate cell survival (Föller et al., 2009).

High energetic demands and auxotrophy by intracellular pathogens present a targetable approach to limit their growth. Drugs typically target pathogen-specific molecules, but due to the risk of selecting and spreading drug-resistant parasites, targeting of host molecules or pathways critical for successful pathogen development is an enticing strategy toward disease control. Host-based interventions have already been proposed against several pathogens, including hepatic and erythrocytic *Plasmodium* stages. For example, host p53 and Bcl-2 (Douglass et al., 2015), heme oxygenase 1 (Pena et al., 2012), erythrocyte G protein (Murphy et al., 2006), and MEK kinases (Sicard et al., 2011) have been suggested as potential targets. This concept is particularly valuable in the context of co-infections where multiple diseases could be tackled at once. The results presented here reveal the host AMPK as a druggable target with the potential to be further explored for antimalarial chemoprophylaxis and/or combination therapies.

EXPERIMENTAL PROCEDURES

Cells, Transfections, and Infections

Cells were infected by adding freshly dissected *P. berghei*, *P. yoelii*, or *P. falciparum* sporozoites and analyzed by immunofluorescence assay or luminescence assay for luciferase-expressing parasites. For AMPKα knockdown, siPOOLs antisense oligonucleotides directed against *prkaa1* and *prkaa2* were used (siTOOLs Biotech). For AMPKα1 overexpression, cells were transiently transfected with pEBG-AMPKα1 plasmid (27632, Addgene) prior to infection.

Mice, Diets, and Treatments

Male C57BL/6 mice were grouped based on body weight, housed four to five per cage, and allowed free access to water and food, except for mice on dietary restriction, which were given daily 60%–70% of the food consumed by the control group. Salicylate treatment was performed by intraperitoneal injection. Mice infections were performed by intravenous (5×10^4 spz per mouse) or intradermal (5×10^3 spz per mouse) injections and analyzed by microscopy on extracted livers or by flow cytometry, respectively. All experiments in animals were approved by the animal ethics committee at Instituto de Medicina Molecular, Lisboa (Portugal) and performed according to national and European regulations.

Statistical Analysis

Statistics were determined with a Student's t or Mann-Whitney U test for comparisons between two conditions and a one-way ANOVA for comparisons involving three or more conditions. Statistical significance was considered for p values below 0.05. The outliers in the boxplots represent 5%–10% of data points. Values in bar graphs are means ± SEM, and data mentioned in the text are means ± SD.

SUPPLEMENTAL INFORMATION

Supplemental Information includes Supplemental Experimental Procedures, five figures, and one table and can be found with this article online at http://dx.doi.org/10.1016/j.celrep.2016.08.001.

AUTHOR CONTRIBUTIONS

Conceptualization, M.M.M. and L.M.-S.; Investigation, M.T.G.R., I.M.V., J.S.-D., P.M., N.G., and L.M.-S.; Writing – Original Draft, M.T.G.R., I.M.V., and L.M.-S.; Writing – Review & Editing, M.T.G.R., I.M.V., M.M.M., and L.M.-S.; Funding Acquisition, M.M.M. and L.M.-S.; Supervision, S.N.B., M.M.M., and L.M.-S.

ACKNOWLEDGMENTS

We would like to thank Benoit Viollet for providing the AMPKα-null MEFs; Ana Parreira for mosquito and sporozoite production; Sandra March and Alex Miller for technical assistance; Rogerio Amino for advice on intradermal injections; and Eliana Real and Elena Baena-Gonzalez for critical reading of the manuscript. This work was supported by European Commission (FP7/2007-2013) grant agreement No. 242095 (EVIMalaR) to L.M.-S. and M.M.M.; and Fundação para a Ciência e Tecnologia (Portugal) through grants

PTDC/SAU-MET/118199/2010 and EXCL/IMI-MIC/0056/2012 to L.M.-S. and M.M.M., respectively. M.M.M. was also supported by the ERC (agreement No. 311502). I.M.V. was sponsored by EMBO LTF 712-2012 and NIH NRSA 5F32AI104252 fellowships.

Received: December 5, 2015
Revised: April 20, 2016
Accepted: July 28, 2016
Published: August 25, 2016

REFERENCES

Albuquerque, S.S., Carret, C., Grosso, A.R., Tarun, A.S., Peng, X., Kappe, S.H., Prudêncio, M., and Mota, M.M. (2009). Host cell transcriptional profiling during malaria liver stage infection reveals a coordinated and sequential set of biological events. BMC Genomics *10*, 270.

Brunton, J., Steele, S., Ziehr, B., Moorman, N., and Kawula, T. (2013). Feeding uninvited guests: mTOR and AMPK set the table for intracellular pathogens. PLoS Pathog. *9*, e1003552.

Crute, B.E., Seefeld, K., Gamble, J., Kemp, B.E., and Witters, L.A. (1998). Functional domains of the alpha1 catalytic subunit of the AMP-activated protein kinase. J. Biol. Chem. *273*, 35347–35354.

Deschermeier, C., Hecht, L.S., Bach, F., Rützel, K., Stanway, R.R., Nagel, A., Seeber, F., and Heussler, V.T. (2012). Mitochondrial lipoic acid scavenging is essential for Plasmodium berghei liver stage development. Cell. Microbiol. *14*, 416–430.

Douglass, A.N., Kain, H.S., Abdullahi, M., Arang, N., Austin, L.S., Mikolajczak, S.A., Billman, Z.P., Hume, J.C., Murphy, S.C., Kappe, S.H., et al. (2015). Host-based prophylaxis successfully targets liver stage malaria parasites. Mol. Ther. *23*, 857–865.

Föller, M., Sopjani, M., Koka, S., Gu, S., Mahmud, H., Wang, K., Floride, E., Schleicher, E., Schulz, E., Münzel, T., and Lang, F. (2009). Regulation of erythrocyte survival by AMP-activated protein kinase. FASEB J. *23*, 1072–1080.

Hardie, D.G. (2014). AMP-activated protein kinase: maintaining energy homeostasis at the cellular and whole-body levels. Annu. Rev. Nutr. *34*, 31–55.

Hardie, D.G., and Pan, D.A. (2002). Regulation of fatty acid synthesis and oxidation by the AMP-activated protein kinase. Biochem. Soc. Trans. *30*, 1064–1070.

Hawley, S.A., Fullerton, M.D., Ross, F.A., Schertzer, J.D., Chevtzoff, C., Walker, K.J., Peggie, M.W., Zibrova, D., Green, K.A., Mustard, K.J., et al. (2012). The ancient drug salicylate directly activates AMP-activated protein kinase. Science *336*, 918–922.

Itoe, M.A., Sampaio, J.L., Cabal, G.G., Real, E., Zuzarte-Luis, V., March, S., Bhatia, S.N., Frischknecht, F., Thiele, C., Shevchenko, A., and Mota, M.M. (2014). Host cell phosphatidylcholine is a key mediator of malaria parasite survival during liver stage infection. Cell Host Microbe *16*, 778–786.

Kalanon, M., Bargieri, D., Sturm, A., Matthews, K., Ghosh, S., Goodman, C.D., Thiberge, S., Mollard, V., McFadden, G.I., Ménard, R., and de Koning-Ward, T.F. (2016). The Plasmodium translocon of exported proteins component EXP2 is critical for establishing a patent malaria infection in mice. Cell. Microbiol. *18*, 399–412.

Kaushansky, A., Ye, A.S., Austin, L.S., Mikolajczak, S.A., Vaughan, A.M., Camargo, N., Metzger, P.G., Douglass, A.N., MacBeath, G., and Kappe, S.H. (2013). Suppression of host p53 is critical for Plasmodium liver-stage infection. Cell Rep. *3*, 630–637.

Kemp, L.E., Yamamoto, M., and Soldati-Favre, D. (2013). Subversion of host cellular functions by the apicomplexan parasites. FEMS Microbiol. Rev. *37*, 607–631.

Kim, J., Kundu, M., Viollet, B., and Guan, K.L. (2011). AMPK and mTOR regulate autophagy through direct phosphorylation of Ulk1. Nat Cell Biol. *13*, 132–141.

Labaied, M., Jayabalasingham, B., Bano, N., Cha, S.J., Sandoval, J., Guan, G., and Coppens, I. (2011). Plasmodium salvages cholesterol internalized by LDL and synthesized de novo in the liver. Cell. Microbiol. *13*, 569–586.

Laderoute, K.R., Amin, K., Calaoagan, J.M., Knapp, M., Le, T., Orduna, J., Foretz, M., and Viollet, B. (2006). 5′-AMP-activated protein kinase (AMPK) is induced by low-oxygen and glucose deprivation conditions found in solid-tumor microenvironments. Mol. Cell. Biol. *26*, 5336–5347.

Mankouri, J., Tedbury, P.R., Gretton, S., Hughes, M.E., Griffin, S.D., Dallas, M.L., Green, K.A., Hardie, D.G., Peers, C., and Harris, M. (2010). Enhanced hepatitis C virus genome replication and lipid accumulation mediated by inhibition of AMP-activated protein kinase. Proc. Natl. Acad. Sci. USA *107*, 11549–11554.

Moreira, D., Rodrigues, V., Abengozar, M., Rivas, L., Rial, E., Laforge, M., Li, X., Foretz, M., Viollet, B., Estaquier, J., et al. (2015). Leishmania infantum modulates host macrophage mitochondrial metabolism by hijacking the SIRT1-AMPK axis. PLoS Pathog. *11*, e1004684.

Moser, T.S., Schieffer, D., and Cherry, S. (2012). AMP-activated kinase restricts Rift Valley fever virus infection by inhibiting fatty acid synthesis. PLoS Pathog. *8*, e1002661.

Mota, M.M., Pradel, G., Vanderberg, J.P., Hafalla, J.C., Frevert, U., Nussenzweig, R.S., Nussenzweig, V., and Rodríguez, A. (2001). Migration of Plasmodium sporozoites through cells before infection. Science *291*, 141–144.

Murphy, S.C., Harrison, T., Hamm, H.E., Lomasney, J.W., Mohandas, N., and Haldar, K. (2006). Erythrocyte G protein as a novel target for malarial chemotherapy. PLoS Med. *3*, e528.

Pena, A.C., Penacho, N., Mancio-Silva, L., Neres, R., Seixas, J.D., Fernandes, A.C., Romão, C.C., Mota, M.M., Bernardes, G.J., and Pamplona, A. (2012). A novel carbon monoxide-releasing molecule fully protects mice from severe malaria. Antimicrob. Agents Chemother. *56*, 1281–1290.

Risco-Castillo, V., Topçu, S., Marinach, C., Manzoni, G., Bigorgne, A.E., Briquet, S., Baudin, X., Lebrun, M., Dubremetz, J.F., and Silvie, O. (2015). Malaria sporozoites traverse host cells within transient vacuoles. Cell Host Microbe *18*, 593–603.

Salcedo-Sora, J.E., Caamano-Gutierrez, E., Ward, S.A., and Biagini, G.A. (2014). The proliferating cell hypothesis: a metabolic framework for Plasmodium growth and development. Trends Parasitol. *30*, 170–175.

Sicard, A., Semblat, J.P., Doerig, C., Hamelin, R., Moniatte, M., Dorin-Semblat, D., Spicer, J.A., Srivastava, A., Retzlaff, S., Heussler, V., et al. (2011). Activation of a PAK-MEK signalling pathway in malaria parasite-infected erythrocytes. Cell. Microbiol. *13*, 836–845.

Singh, A.P., Buscaglia, C.A., Wang, Q., Levay, A., Nussenzweig, D.R., Walker, J.R., Winzeler, E.A., Fujii, H., Fontoura, B.M., and Nussenzweig, V. (2007). Plasmodium circumsporozoite protein promotes the development of the liver stages of the parasite. Cell *131*, 492–504.

Singhal, A., Jie, L., Kumar, P., Hong, G.S., Leow, M.K., Paleja, B., Tsenova, L., Kurepina, N., Chen, J., Zolezzi, F., et al. (2014). Metformin as adjunct antituberculosis therapy. Sci. Transl. Med. *6*, 263ra159.

Steinberg, G.R., Dandapani, M., and Hardie, D.G. (2013). AMPK: mediating the metabolic effects of salicylate-based drugs? Trends Endocrinol. Metab. *24*, 481–487.

Sturm, A., Amino, R., van de Sand, C., Regen, T., Retzlaff, S., Rennenberg, A., Krueger, A., Pollok, J.M., Menard, R., and Heussler, V.T. (2006). Manipulation of host hepatocytes by the malaria parasite for delivery into liver sinusoids. Science *313*, 1287–1290.

World Health Organization. (2015). World Malaria Report 2015. http://www.who.int/malaria/publications/world-malaria-report-2015/report/en/.

Cell Reports, Volume *16*

Supplemental Information

Host AMPK Is a Modulator of *Plasmodium* Liver Infection

Margarida T. Grilo Ruivo, Iset Medina Vera, Joana Sales-Dias, Patrícia Meireles, Nil Gural, Sangeeta N. Bhatia, Maria M. Mota, and Liliana Mancio-Silva

SUPPLEMENTAL FIGURES

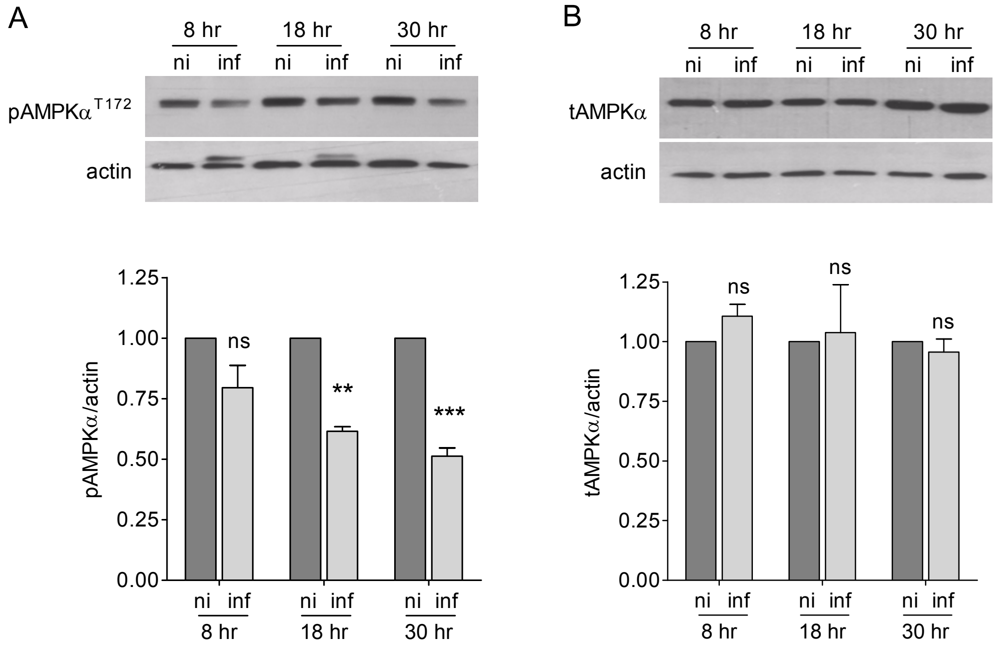

Figure S1. Related to Figure 1. Western blot analysis of lysates from *P. berghei* FACS-enriched infected (inf) and non-infected (ni) Huh7 cells harvested at 8, 18 and 30 hr post infection. Representative blots probing pAMPKα^{T172} (A) and total AMPKα (B). Stripping of the phospho antibody from the membranes was not efficient, therefore the same samples were loaded and analyzed in a separate membrane. Quantitative analysis of pAMPKα^{T172} and total AMPKα from 3 independent experiments (mean±SEM) is shown in the bottom panels. ns, non-significant; **$p<0.01$; ***$p<0.001$.

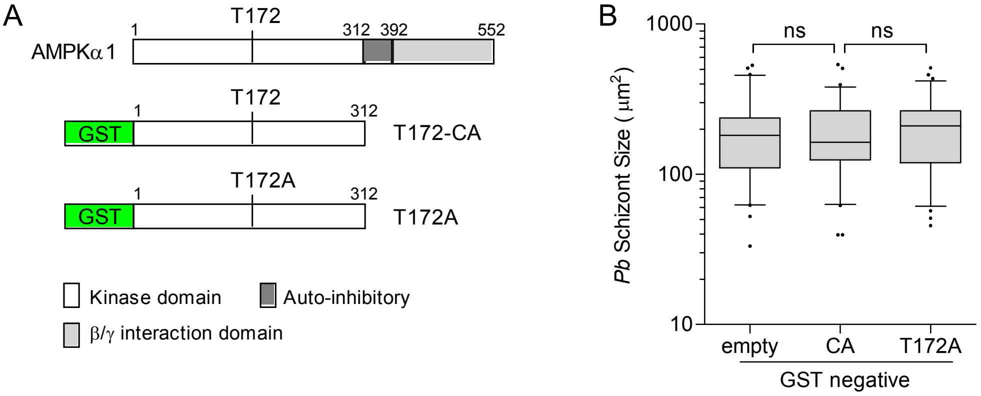

Figure S2. Related to Figure 2. (A) Schematic of the full-length AMPKα1 and its three domains: the catalytic domain (residues 1-312) containing the T172 phosphorylation site, the auto-inhibitory domain (312-392), and the C-terminal βγ interaction domain (392-552). Representation of the truncated constitutively active (CA) is shown below. The mutant construct (T172A) cannot be phosphorylated due to the replacement of the threonine to an alanine, and is used as negative control. The AMPKα1 CA and T172A proteins contain a N-terminal GST tag. (B) Quantification of parasite size (area) in Huh7 cells expressing the CA AMPKα1, T172A AMPKα1, or GST only (empty plasmid) in GST negative cells (50-70 parasites analyzed per condition). The mean±SD are as follows: 195.7±108.7μm^2, empty; 213.2±106.4μm^2, CA; 196.7±104.6μm^2, T172A. Graph shown is representative of 3 independent experiments. ns, non-significant.

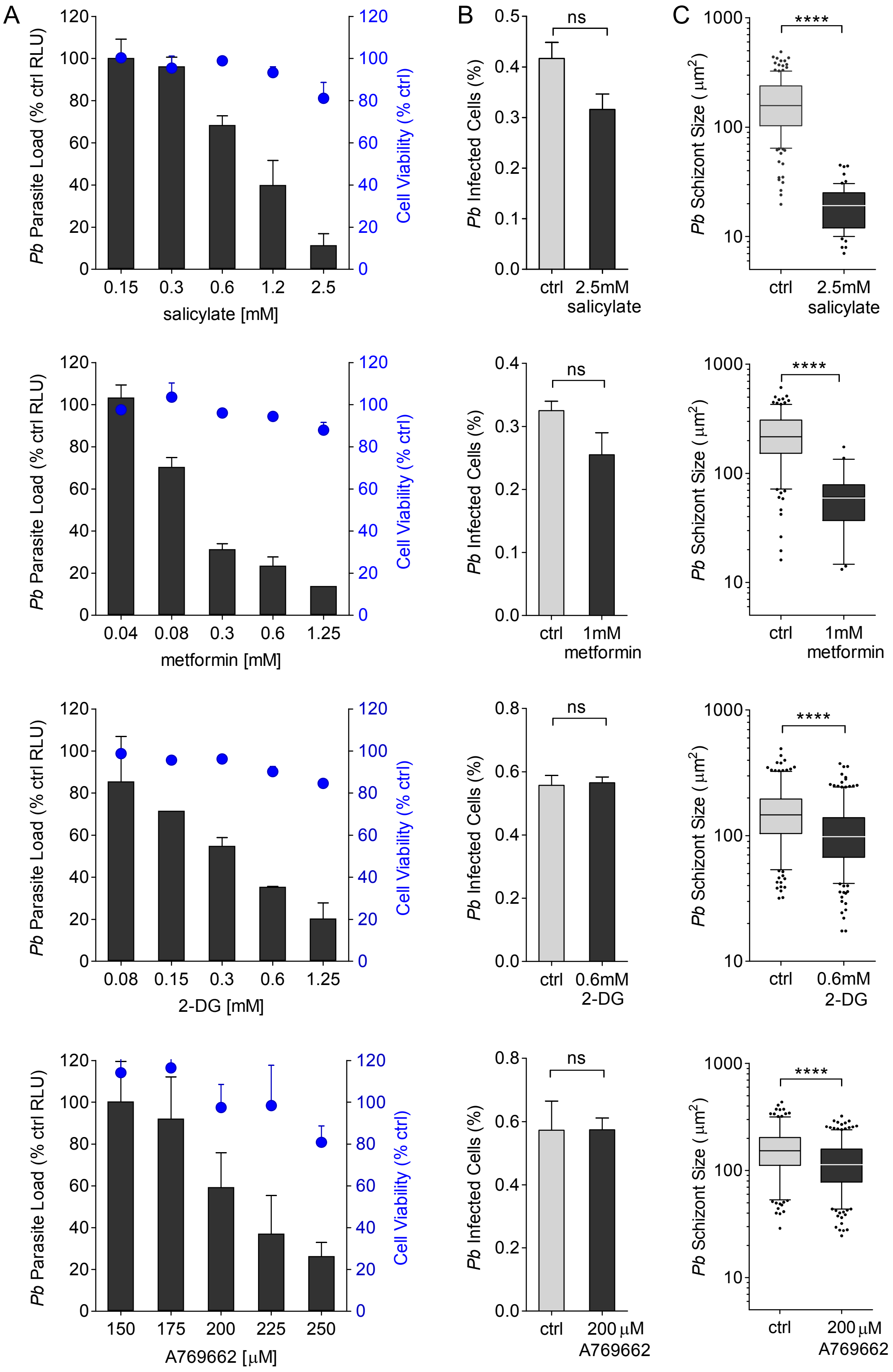

Figure S3. Related to Figure 3. Dose-dependent effect of various AMPK agonists: salicylate, metformin, 2-deoxy-D-Glucose (2-DG), and A769662 on *P. berghei* infection of Huh7 cells. (A) Parasite load at 48 hr post infection was measured via luminescence and is plotted as bar graphs (left y-axis) and host cell viability (right y-axis, blue) is plotted as blue data points above each bar. Values are mean±SEM from 2-3 independent experiments. EC_{50} values are in Table S1 and were determined by GraphPad Prism using non-linear regression variable slope (normalized) analysis. (B-C) Quantification of luciferase-expressing *P. berghei* schizont numbers (B) and size (C) by microscopic analysis. Data are representative of 2-3 independent experiments.

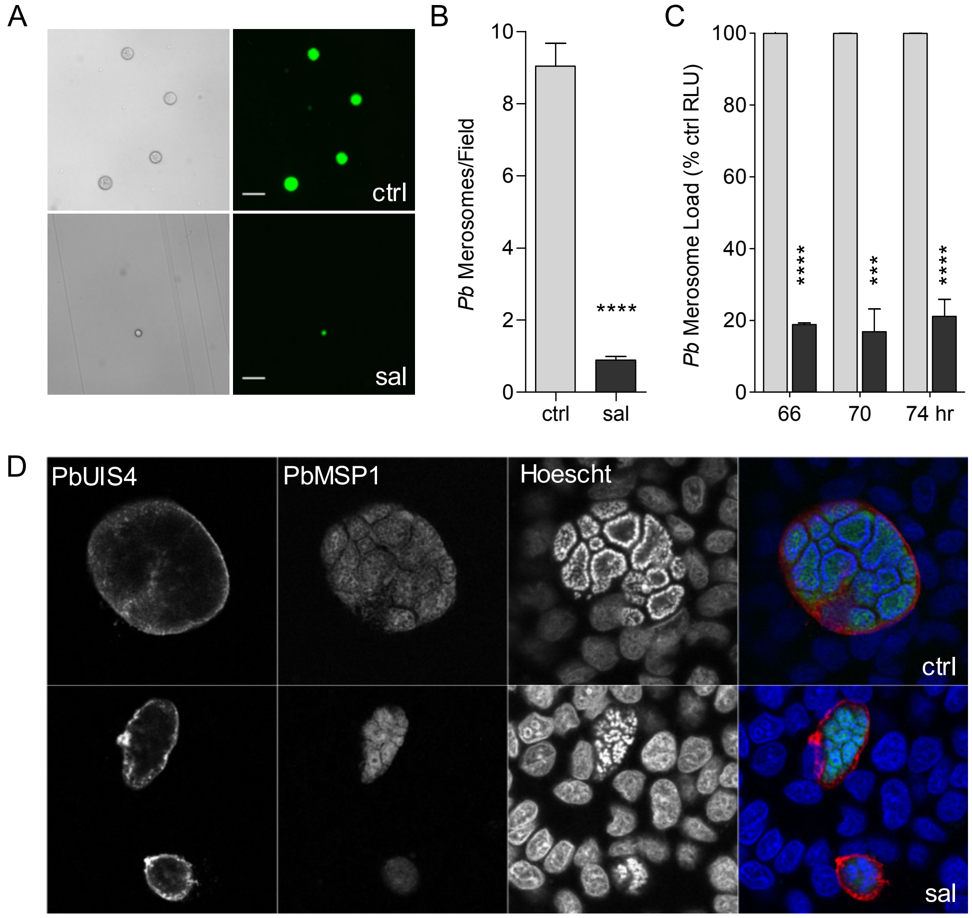

Figure S4. Related to Figure 3. Merosome analysis.
(A-B) Live GFP and bright field representative images (A) and quantification (B) of *P. berghei* detached merosomes at 66 hr after infection of HepG2 cells and treatment with 2.5mM salicylate (sal) or the vehicle control (ctrl, water). Similar results were observed upon treatment with A769662 (*data not shown*). Scale bars, 20µm. ****$p<0.0001$.
(C) Luminescence levels from detached merosomes at multiple time-points after infection of HepG2 cells with luciferase-expressing *P. berghei* parasites under salicylate treatment (2.5mM). Relative luminescence values (RLU) were measured at 66, 70, and 74 hr. The bars are mean±SEM normalized to the correspondent untreated control, from 3 independent experiments. ***$p<0.001$; ****$p<0.0001$.
(D) HepG2 cells at 66 hr after infection, treated with vehicle (ctrl) or 2.5mM salicylate (sal), probing with anti-PbUIS4 (red) and anti-MSP1 (green) antibodies. DNA is stained with Hoechst (blue). Images acquired on a confocal microscope with a 40x magnification.

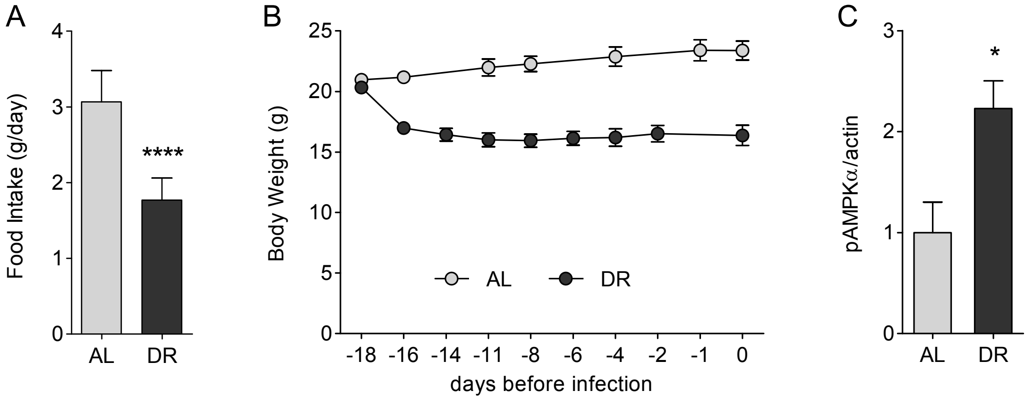

Figure S5. Related to Figure 4. Average food intake (A) and body weight change (B) in male C57BL/6 mice fed *ad libitum* (AL) or in mice under dietary restriction (DR) regimen. Mice on DR were given daily 60-70% of the food consumed by the AL group, for 2 to 3 weeks prior to infection to avoid stress effects and allow for weight stabilization. To determine whether DR was working as expected we monitored body weight every 2-3 days prior to infection. DR mice show approximately 20% loss of the initial body weight. Values are mean±SD (5 mice/group). Representative experiment of 3 independent DR experiments. (C) Quantification of pAMPKα^{T172} in AL and DR mice. Values in the bars are mean±SEM (3 mice/group). *p<0.05; ****p<0.0001.

Table S1. Related to Figure 3. AMPK agonists used in the study.

**Compound**	**Mechanism of AMPK activation**	**References**	***Pb* EC$_{50}$ (µM)**
Salicylate	Direct binding to AMPKβ1 subunit	(Hawley et al., 2012)	950±11
A769662	Direct binding to AMPKβ1 subunit	(Goransson et al., 2007)	214±29
Metformin	Direct inhibition of complex 1 of the respiratory chain	(Owen et al., 2000)	156±70
2-Deoxy-D-glucose	Inhibition of glycolysis	(Woodward and Hudson, 1954)	273±53

SUPPLEMENTAL EXPERIMENTAL PROCEDURES

Chemicals

Salicylate (71945), metformin (D150959), and 2-Deoxy-D-glucose (D6134), were obtained from Sigma. A769662 (171258) was purchased from Calbiochem. The stock solutions were as follows: 1M salicylate (dH_2O); 50mM metformin (dH_2O); 100mM 2-Deoxy-D-glucose (dH_2O); 100mM A769662 (DMSO).

Parasite Lines

P. berghei ANKA expressing GFP (259cl2), RFP (733cl1), and Luciferase (676m1cl1) parasite lines were obtained from the Leiden Malaria Research Group (www.pberghei.eu). *P. yoelii* 17X NL parasites were obtained through the MR4 (www.mr4.org). *P. berghei* and *P. yoelii* sporozoites were isolated from salivary glands of *Anopheles stephensi*, bred at Instituto de Medicina Molecular (Lisboa, Portugal). *P. falciparum* sporozoites were obtained by dissection of salivary glands from infected *Anopheles gambiae* mosquitoes obtained from the insectary at Johns Hopkins School of Public Health (Baltimore, USA) or Sanaria Inc. (USA).

Cells Lines and Infections

Cells were cultured in medium supplemented with Fetal Bovine Serum (FBS), 50 µg/mL Penicillin/Streptomycin, and 2mM Glutamine (all Gibco) at 37°C with 5% CO_2. RPMI medium was also supplemented with 0.1mM non-essential amino acids. Cells were always infected 24 hr post seeding. At time of infection, medium was removed and freshly dissected *P. berghei* or *P. yoelii* sporozoites in supplemented medium containing Fungizone (1µg/mL, Gibco) were added to the wells, followed by a 5-minute centrifugation at 3000 rpm. In all assays, unless stated otherwise, medium was changed 2 hr after infection either to add fresh, non-treated medium, or to add different compounds, always supplemented with Fungizone (Gibco). Details of cell lines, numbers of cells seeded, and sporozoites used for different experiments can be found in the table below.

Micropatterned coculture (MPCC) preparation and *P. falciparum* infection were carried out as described previously (Khetani and Bhatia, 2008; March et al., 2013). Briefly, glass-bottom 96-well plates were coated homogenously with rat tail type I collagen (50 µg/ml) and subjected to soft-lithographic techniques to pattern the collagen into microdomains of 500-µm islands that mediate selective hepatocyte adhesion. To create MPCCs, cryopreserved primary human hepatocytes (Life Technologies) were pelleted by centrifugation at 100 × g for 6 min at 4°C, assessed for viability using trypan blue exclusion, and seeded on collagen-micropatterned plates. Each well contained approximately 1×10^4 hepatocytes organized in colonies of 500 µm in serum-free DMEM with Penicillin/Streptomycin. The cells were washed in complete medium 3 hr post seeding, and the medium was switched to human hepatocyte culture medium. One day after seeding, 75×10^3 freshly dissected *P. falciparum* sporozoites were added to each well. The cells were washed twice 3 hr after sporozoite addition, and 7×10^3 3T3-J2 murine embryonic fibroblasts per well were seeded in human hepatocyte culture medium containing 2 mM salicylate or control vehicle. 5 days post infection, cells were fixed and analyzed by immunofluorescence assay.

Mouse Primary Hepatocytes

Primary hepatocytes were isolated from livers of adult C57BL/6 male mice following an adaptation of the previously described two-step *in situ* perfusion method (Liehl et al., 2014; Seglen, 1976). Briefly, mice were sacrificed by CO_2 inhalation and immediately opened to begin the process of perfusion. The portal vein was cannulated with a 26 g needle and liver was perfused with 30-40 mL of Liver Perfusion Medium (LPM, Gibco) at 37°C followed by digestion with 30-40 mL of Liver Digest Medium (LDM, Gibco) at a flow rate of 7-9 mL/min, controlled by a peristaltic pump. Outflow drain was achieved by cutting the inferior vena cava. Following perfusion and digestion, the liver was carefully removed and placed on a cell culture dish containing 10 mL of LDM and the capsule membrane was carefully peeled away with fine tweezers. The liver was gently shaken to release any loose cells. The cell suspension, containing primary hepatocytes, non-parenchymal cells (NPCs), and dead cells, was serially passed through a 100 µm strainer followed by a 70 µm strainer and was washed twice with 30 mL of 4% FBS supplemented William's E Medium (Gibco) at 30 g for 3 minutes at 20°C. The hepatocyte fraction was then purified to remove any contaminating NPCs and dead cells by layering over a 60% Percoll gradient (GE Healthcare) followed by centrifugation for 20 minutes, 750 g at 20°C with no break. Purified hepatocyte pellet was washed twice with William's E Medium, cells were counted and assessed for viability with Trypan blue. Viable hepatocytes were plated on collagen-coated plates and allowed to settle and attach overnight and infected as described above for hepatoma cells.

**Cells**	**Medium**	**FBS**	**Plate**	**Cells/well**	**Spz**	**Parasite**	**Assay**
Huh7	RPMI	10%	96	1×10^4	8×10^3	Luciferase	Luminescence
			24	5×10^4	5×10^4	GFP	Microscopy
			24	5×10^4	5×10^4	Luciferase	Microscopy
			24	5×10^4	75×10^3	GFP	Transfection
			24	3×10^4	5×10^4	GFP	siPools KD
		20%	24	8×10^4	7×10^4	GFP/RFP	Sorting
HepG2	DMEM	10%	24	5×10^4	5×10^4	Luciferase	Luminescence
MEFs wt	DMEM	10%	24	3×10^4	5×10^4	GFP	Microscopy
MEFs AMPKα-null	DMEM	10%	24	4×10^4	5×10^4	GFP	Microscopy
Mouse primary hepatocytes	William's E	4%	24	12×10^4	5×10^4	GFP	Microscopy
Hepa1-6	DMEM	10%	24	4×10^4	$3\text{-}4\times10^4$	GFP	Microscopy

Cell Sorting

Huh7 cells in 24-well plates were infected with sporozoites as described in the table above. Cells were harvested at 2 hr after infection by trypsinization, passed through a 70 µm filter, washed with RPMI supplemented with 20% FBS and centrifuged 1200 rpm at room temperature for 5 minutes. Cell pellet was resuspended in RPMI 20% FBS and sorted with BD FACSARIA III sorter (BD Biosciences). Cells were sorted with a gate on the GFP/RFP+ cells (infected) and a gate on the GFP/RFP- population (non-infected), at the same flow rate, and collected into 1 mL of RPMI 20% FBS. Collected samples were kept at 4°C and seeded at a 1:1 ratio (infected:non-infected) onto collagen-coated 48-well plates (5×10^4 cells/well) in RPMI 20% FBS and Fungizone. Cells were harvested at 8 hr, 18 hr, or 30 hr post infection and processed for Western blot as described below.

Western Blotting

Prior to cell lysis, medium was removed and cells were washed with ice-cold PBS. 80 µl of lysis buffer were immediately added and incubated on ice for 15 min. Cells were lysed in ice-cold RIPA buffer (150 mM Sodium Chloride, 1% triton X-100, 0.5% Sodium Deoxycholate, 0.1% SDS and 50 mM Tris pH 8.0) containing protease and phosphatase inhibitors (Complete and phosSTOP, Roche). Cells were harvested with a cell scraper and centrifuged at 14 000 rpm, 4°C for 10 minutes to pellet non-soluble cell material. The supernatant containing soluble fraction was collected and kept at -80°C or processed immediately.

Livers were homogenized in ice-cold lysis buffer (50 mM Hepes, 150 mM NaCl, 10 mM NaF, 1 mM Sodium pyrophosphate, 0.5 mM EDTA, 1 mM DTT, 1% triton, 1 mM Na_3VO_4, 250 mM Sucrose, protease inhibitor cocktail and phosphatase inhibitors).

Total protein content was measured by Bradford Assay (Biorad), according to manufacturer's instructions. 15 µg of total cell or 50 µg of total liver lysates were resolved on either 8% SDS-PAGE or Any kD mini-protean precast gels (Biorad) and transferred to a nitrocellulose membrane using standard wet transfer with 1x Tris-Glycine buffer containing 20% methanol for 2 hr at 100 V constant or were transferred using iBlot gel Transfer stacks (ThermoFisher). Membranes were blocked in TBS-5% BSA Tween 0.2% for 1 hr at room temperature and incubated with primary antibodies overnight at 4°C.

pACC and pAMPK were detected using rabbit anti-phosho-ACC^{S79} (mAb D7D11, 1:1000) and rabbit anti-phospho-$AMPK\alpha^{T172}$ (mAb 40H9, 1:1000), respectively (both from Cell Signaling Technology, CST). Total AMPKα was detected using rabbit antibody (CST mAb D63G4, 1:1000). Anti-GST rabbit polyclonal antibody (Abcam ab9085, 1:500) was used to detect Glutathione S-Transferase. Anti-actin (Sigma-Aldrich A2066, 1:1000) rabbit antibody was used as loading control (incubation for 1 hr at room temperature). Horseradish peroxidase (HRP)-conjugated goat anti-rabbit IgG, Fc fragment specific and HRP-conjugated goat anti-mouse IgG, light chain specific (both from Jackson ImmunoResearch) were used as secondary antibodies.

Luminescence Assay

Infected cells were washed with PBS at 48 hr after infection and lysed in 75 µl of 1x Firefly lysis buffer from Firefly Luciferase Assay Kit (Biotium). Plates were shaken for 20 minutes and then centrifuged for 5 minutes, 3000 rpm. 50 µl of D-Luciferin dissolved in the kit buffer were added to 30 µl of total lysate in white 96-well plates and luciferase activity was measured using a multiplate reader (Infinite 200M, Tecan). Cell viability was assayed by Alamar Blue assay (Invitrogen) using the manufacturer's protocol, following an incubation of 90 minutes at 37°C.

For quantification of detached merosomes, medium was replenished at 48 hr post infection with 50 µl of complete medium. At 66-74 hr, supernatants were collected and lysed with 12.5 µl of 5x Firefly lysis buffer and incubated at room temperature for 10 minutes with gentle shaking. The whole lysate (60 µl) was transferred to a white plate as mentioned above, and luciferase activity was measured after adding 100 µl (1 mg/mL) of D-luciferin dissolved in firefly luciferase assay buffer.

Live Fluorescence Imaging and Quantification of Detached Merosomes

Supernatant (~500 µl) containing detached merosomes from HepG2 cells infected with *P. berghei* expressing GFP (at 66 hr post infection) were collected. Merosomes were pelleted at 1200 rpm for 5 minutes, supernatant removed, and pellet was resuspended in 20 µl of complete DMEM. Merosomes were quantified by loading 10 µl onto a hemocytometer and visualized with an upright widefield fluorescence microscope as described below. At least 10 fields were imaged and quantified per condition. The acquired images were then analyzed on ImageJ (http://imagej.nih.gov/ij/) to quantify the size of the parasite, which is the area of the parasite as determined by fluorescence intensity. Counts were determined as number of merosomes counted per field at 10x magnification.

Immunofluorescence Assay

Infected cells on coverslips (in triplicate) were fixed in 4% Paraformaldehyde (PFA) for 10 minutes, permeabilized in PBS 0.1% Triton-100 for 10 minutes and blocked in PBS 0.1% Triton-100 and 1% BSA for 30 minutes. Primary antibody staining was performed in blocking solution for 1 hr with the following antibodies: goat anti-PbUIS4 (1:1000), mouse anti-PbHSP70 (2E6, 1:200), rabbit anti-PbMSP1 (1:450), mouse anti-PfHSP70 (clone 4C9, 1:200), rabbit anti-GST (Abcam ab9085, 1:400). Primary antibodies were detected using several AlexaFluor-conjugated antibodies (Molecular Probes/Invitrogen): donkey anti-goat 568 (1:400), donkey anti-mouse 488 or 647 (1:400), donkey anti-rabbit 488 (1:400). Coverslips were mounted in Fluoromount-G (Southern Biotech).

Livers were fixed with 4% PFA for 2 hr and sliced into 50 µm-thick sections using the Vibratome VT 1000 S (Leica). Liver sections were permeabilized and blocked in PBS 1% BSA and 0.3% Triton-100 for 45 minutes, and incubated with goat anti-PbUIS4 (1:1000) for 1 hr. After washing in PBS, liver sections were incubated with donkey anti-goat conjugated to Alexa Fluor 555 (1:400) and Hoechst (1:1000) for another 1 hr and mounted in Fluoromount-G. All incubations were performed at room temperature.

Imaging Analysis

30 fields in each coverslip (triplicates) were randomly acquired using the MetaMorph software (Molecular Devices) with an inverted wide-field fluorescence microscope, Axiovert 200M (Zeiss) with a 20x magnification. For transfected cells and liver sections, acquisition was performed by non-random identification of parasites in the entire coverslip. The acquired images were then analyzed on ImageJ (http://imagej.nih.gov/ij/) to quantify parasite size, determined as the area of parasite defined by the staining of PbUIS4 or HSP70. We established a minimum size cut-off of 10 μm^2. The total number of cells was estimated by the total number of nuclei per image, and used to obtain the percentage of infected cells.

Illustrative images of *P.berghei* schizonts (Figure S4) and infected liver sections (Figure 4) were taken on confocal microscope, LSM 510 Meta (Zeiss) and *P. falciparum* liver schizonts were imaged on Nikon A1R Ultra-Fast Spectral Scanning Confocal Microscope (Figure 3F). Live GFP imaging of merosomes were taken on fluorescence microscope Leica DM5000B (Figure 3H, S4A).

siRNA Knockdown

Knockdown experiments were performed in 24-well plates containing coverslips for microscopy using a reverse transfection protocol. Huh7 cells were trypsinized, resuspended in antibiotic-free complete RPMI, counted, and prepared for seeding at a final concentration of 75×10^3 cells/mL. Meanwhile, siRNA complexes, targeting multiple sites on a single transcript to increase specificity and avoid off-targets (Hannus et al., 2014), were prepared following manufacturer's protocol. Briefly, siPool oligonucleotides (siTools Biotech GmbH, Martinsried, Germany) targeting human *prkaa1a* (NM_006251) and *prkaa2a* (NM_006252), or control siPool were pre-mixed in 100 µl of Opti-MEM (Gibco) with 0.2 µl of Lipofectamine RNAiMax (Invitrogen) transfection reagent for a final concentration of 3 nM siPool/well. Complexes of siPool/RNAiMax were aliquoted (100 µl) into each well and incubated at room temperature for 15 minutes. Cells were then seeded on top of the complexes in a total volume of 400 µl at seeding density of 3×10^4 cells/well. 24 hr post transfection, medium was replenished with complete RMPI containing antibiotics. 48 hr post transfection, cells were harvested for assessment of knockdown efficiency by Western blot and/or infected with 5×10^4 *P. berghei* sporozoites/well for microscopic analysis of parasite infection.

Site Directed Mutagenesis and Transfection

For AMPKα overexpression in cells, the following plasmids were used: empty plasmid (Tanaka 1995; pEBG; 22227, Addgene), pEBG-AMPKα1 (1-312) plasmid (Crute et al., 1998; Egan et al., 2011; 27632, Addgene), and pEBG-AMPKα1 was mutated by site-directed mutagenesis to make T172A. Site-directed mutagenesis was performed with the QuickChange II XL Site-Directed Mutagenesis Kit (Agilent Technologies) according to manufacturer's instructions and verified by sequencing. Primer pairs are as follows: GGTGAATTTTTAAGAGCGAGCTGTGGCTCGCCCAATTATGCTGC, and GCAGCATAATTGGGCGAGCCACAGCTCGCTCTTAAAAATTCACC.

Cells were reverse transfected with the AMPKα CA, AMPKα T172A and empty plasmids, using FuGENE 6 Transfection Reagent (Promega). DNA was added to the transfection reagent in a ratio of 3:1, to a final amount of 0.5 µg of DNA/well. Cells were infected or lysed (for Western blot) 24 hr after transfection.

Flow Cytometry

The percentage of infected red blood cells (parasitemia) at 72 hr after injection of GFP-expressing sporozoites into mice was determined by flow cytometry (LSR Fortessa, BD Biosciences). A drop of blood was collected from the mouse tail into ~500 µl PBS, and 1-2×10^6 cells per mouse were analyzed (medium flow speed). Cells were selected on the basis of their size by gating first on FSC and SSC and, subsequently, on FITC (green) and PE (red) channels. The GFP-expressing parasites were detected in the FITC channel. False GFP positive cells (red blood cell's auto-fluorescence) were eliminated by plotting FITC against PE. The analysis was performed by using the FlowJo software (TreeStar, USA).

SUPPLEMENTAL REFERENCES

Crute, B.E., Seefeld, K., Gamble, J., Kemp, B.E., and Witters, L.A. (1998). Functional domains of the alpha1 catalytic subunit of the AMP-activated protein kinase. The Journal of biological chemistry *273*, 35347-35354.

Egan, D., Kim, J., Shaw, R.J., and Guan, K.L. (2011). The autophagy initiating kinase ULK1 is regulated via opposing phosphorylation by AMPK and mTOR. Autophagy *7*, 643-644.

Goransson, O., McBride, A., Hawley, S.A., Ross, F.A., Shpiro, N., Foretz, M., Viollet, B., Hardie, D.G., and Sakamoto, K. (2007). Mechanism of action of A-769662, a valuable tool for activation of AMP-activated protein kinase. The Journal of biological chemistry *282*, 32549-32560.

Hannus, M., Beitzinger, M., Engelmann, J.C., Weickert, M.T., Spang, R., Hannus, S., and Meister, G. (2014). siPools: highly complex but accurately defined siRNA pools eliminate off-target effects. Nucleic acids research *42*, 8049-8061.

Hawley, S.A., Fullerton, M.D., Ross, F.A., Schertzer, J.D., Chevtzoff, C., Walker, K.J., Peggie, M.W., Zibrova, D., Green, K.A., Mustard, K.J.*, et al.* (2012). The ancient drug salicylate directly activates AMP-activated protein kinase. Science *336*, 918-922.

Khetani, S.R., and Bhatia, S.N. (2008). Microscale culture of human liver cells for drug development. Nature biotechnology *26*, 120-126.

Liehl, P., Zuzarte-Luis, V., Chan, J., Zillinger, T., Baptista, F., Carapau, D., Konert, M., Hanson, K.K., Carret, C., Lassnig, C.*, et al.* (2014). Host-cell sensors for Plasmodium activate innate immunity against liver-stage infection. Nature medicine *20*, 47-53.

March, S., Ng, S., Velmurugan, S., Galstian, A., Shan, J., Logan, D.J., Carpenter, A.E., Thomas, D., Sim, B.K., Mota, M.M.*, et al.* (2013). A microscale human liver platform that supports the hepatic stages of Plasmodium falciparum and vivax. Cell host & microbe *14*, 104-115.

Owen, M.R., Doran, E., and Halestrap, A.P. (2000). Evidence that metformin exerts its anti-diabetic effects through inhibition of complex 1 of the mitochondrial respiratory chain. The Biochemical journal *348 Pt 3*, 607-614.

Seglen, P.O. (1976). Preparation of isolated rat liver cells. Methods in cell biology *13*, 29-83.

Woodward, G.E., and Hudson, M.T. (1954). The effect of 2-desoxy-D-glucose on glycolysis and respiration of tumor and normal tissues. Cancer research *14*, 599-605.
